# Integration of Morphological and Genome-Wide SNP Data Reveals Regional Diversity in Thai Swamp Buffalo

**DOI:** 10.3390/ani16172713

**Published:** 2026-09-01

**Authors:** Thanwarat Phophantool, Monchai Duangjinda, Vibuntita Chankitisakul, Wootichai Kenchaiwong, Kecha Kuha, Kitsanathon Sintala, Goonlapat Photikanit, Wuttigrai Boonkum

**Affiliations:** 1 Department of Animal Science, Faculty of Agriculture, Khon Kaen University, Khon Kaen 40002, Thailand; thanwarat.pho@kkumail.com (T.P.); vibuch@kku.ac.th (V.C.); wuttbo@kku.ac.th (W.B.); 2 Network Center for Animal Breeding and Omics Research, Faculty of Agriculture, Khon Kaen University, Khon Kaen 40002, Thailand; wootken@gmail.com; 3 Faculty of Veterinary Sciences, Mahasarakham University, Maha Sarakham 44150, Thailand; 4 Faculty of Agriculture, Ubon Ratchathani Rajabhat University, Ubon Ratchathani 34000, Thailand; kecha.k@ubru.ac.th; 5 Department of Animal Science and Fisheries, Faculty of Agricultural Science and Technology, Rajamangala University of Technology Lanna, Nan 55000, Thailand; kitsanathon@gmail.com; 6 Bureau of Animal Breeding, Department of Livestock Development, DLD Government Complex, Pathum Thani 12000, Thailand; goonlapat.p@dld.go.th

**Keywords:** *Bubalus bubalis*, swamp buffalo, morphology, SNP, genetic structure, genomic admixture

## Abstract

Thai swamp buffalo (Bubalus bubalis) are valuable animal genetic resources, but their regional diversity remains incompletely characterized. Morphological records were obtained from 799 buffaloes, and 474 individuals were genotyped using the 90K Axiom^®^ Buffalo SNP Genotyping Array (Thermo Fisher Scientific, Applied Biosystems™, Santa Clara, CA, USA). Qualitative traits included coat color, horn shape, chevron pattern, and hair-whorl distribution, while quantitative characterization covered 30 body measurements. After excluding missing traits, 30 traits adjusted for sex, age, province, and farm from 768 animals were used for morphology-based analyses, which identified regional phenotypic differences but also substantial overlap, consistent with the influence of feeding, management, environment, and local selection on body conformation. Genome-wide SNP analyses indicated a broadly shared genetic background with detectable regional structure, and runs of homozygosity revealed regional differences in genomic autozygosity. The Lower South showed a comparatively strong recurrent-ROH pattern, whereas inference for the Upper South requires caution because of its small genomic sample. Overall, integrating morphology and genome-wide SNP data improves regional characterization, but data-dependent statistics, population-structure estimates, and conservation implications should be interpreted while regarding small sampling and unbalanced data.

## Simple Summary

Thai swamp buffalo are important to farming, culture, and rural livelihoods, but regional differences remain incompletely characterized. We recorded qualitative traits and body measurements from 799 buffaloes and examined genome-wide Single Nucleotide Polymorphism (SNP) data from the 474 genotyped subset sampled across seven regions of Thailand. Morphological traits adjusted for age, sex, and locations differed among regional populations but may also reflect feeding, management, environment, and local selection. Genome-wide analyses indicated shared ancestry together with detectable regional structure, particularly in the southern populations. Because genomic sample sizes were unbalanced among regions, conclusions for the smallest regional sample should be interpreted cautiously. These findings support integrated phenotypic and genomic characterization for conservation planning and future validation in larger, more balanced datasets.

## 1. Introduction

The Thai swamp buffalo (*Bubalus bubalis*) has long been a valuable animal genetic resource in Thai agricultural and rural societies. Its strength, endurance, and adaptation to humid tropical environments have made it a major source of draft power for rice cultivation, particularly plowing and harrowing. Beyond their role in farming, buffalo also have cultural importance, shown in traditional racing festivals that reflect local identity and the close ties between rural communities and their native livestock [1]. However, agricultural mechanization has greatly reduced the role of buffaloes as draft animals. Together with the loss of grazing areas, degradation of natural forage and swamp habitats, uncontrolled crossbreeding, shortages of quality breeding stock, limited reproductive and conservation technologies, and gaps in management knowledge, this has contributed to a continuing decline in the national buffalo population [1,2]. Across Southeast Asia, including Thailand, swamp buffaloes remain an important reservoir of genetic diversity shaped by diverse habitats, from floodplains and rice-based agroecosystems to upland areas [1,3]. Without effective conservation planning, declining population sizes may accelerate genetic erosion and the loss of distinct regional genetic resources [4,5].

Studies on buffalo diversity commonly combine phenotypic characterization with molecular markers [4,5,6]. Morphological traits allow visual and measurable identification in the field, while genetic data offers clearer resolution of ancestry, gene flow, and population structure [5,6,7]. Qualitative descriptors often include horn shape and orientation, coat or skin color, chevron markings, and hair whorl patterns [4]. However, these traits may be influenced by age, management, and environment and may not fully reflect the genetic structure [4,5]. However, morphology alone can misrepresent population history because phenotypic plasticity and convergent adaptation may produce similar appearances in genetically distinct populations [4,5,6].

Before the genomic era, nuclear microsatellites were widely used to quantify within-population diversity, differentiation, and relationships among Asian swamp and river buffalo populations [7,8]. Mitochondrial D-loop, cytochrome b, COI, and complete mitogenomes subsequently revealed maternal haplotype diversity and geographic structure across Southeast Asian swamp buffalo [3,9,10]. These studies established broad regional lineages but provided limited resolution of recent admixture and fine-scale nuclear structure.

Genomic studies using genome-wide SNP markers are relatively new in buffalo research because high-density buffalo-specific SNP arrays have only become available in the past decade [11]. In addition, earlier work was limited by the poor transferability of bovine SNP chips. Genome-wide SNP data from arrays or sequencing now provide higher-resolution estimates of the population structure in Asian buffalo [6,7,12,13,14]. These data support more precise inferences of admixture, genetic drift, effective population size, and fine-scale differentiation [7,13,14]. Genomic analyses commonly integrate PCA, model-based clustering, and phylogenetic or distance-based reconstruction to clarify the relationships among swamp buffalo populations across China, Indochina, and island Southeast Asia, as well as their connections to river buffalo lineages [6,7,13,14].

Systematic documentation of qualitative and quantitative morphology in Thai and Southeast Asian swamp buffalo is still limited, and earlier genetic studies relied mainly on low-resolution markers such as microsatellites and mitochondrial DNA, which cannot fully resolve population structure. Recent advances in buffalo-specific SNP arrays now allow high-resolution analysis of genetic diversity and ancestry patterns. By comparing morphology, morphometrics, and genome-wide SNP data, this study aims to demonstrate how SNP based genomic analysis provides a clearer and more powerful framework for resolving regional genetic relationships and admixture in Thai swamp buffalo.

## 2. Materials and Methods

### 2.1. Animal

This study characterized 799 swamp buffaloes sampled from 21 provinces and 30 participating farmers across seven regional populations of Thailand. Regions were defined based on geographic distribution and local production contexts (Table 1).

### 2.2. Data Collection

This study was approved by the Institutional Animal Care and Use Committee (IACUC), Khon Kaen University, in accordance with the National Research Council of Thailand guidelines (Approval No. IACUC-KKU-64/67; 3 June 2024). Buffaloes with verified local history were sampled across the seven regions (Table 1). Animals were managed under village-based and farm-specific feeding, grazing, housing, and husbandry systems that could not be standardized across locations. These conditions were therefore treated as potential sources of environmental variation when interpreting morphological traits. According to pedigree, records were not available from farmers, therefore, avoiding relative sampling was purposively selected based on farmers’ knowledge. Morphological and body-conformation measurements were collected, and blood samples were obtained for DNA extraction and SNP genotyping across seven regional populations, as detailed below.

#### 2.2.1. Morphological Data

**Figure 1:**
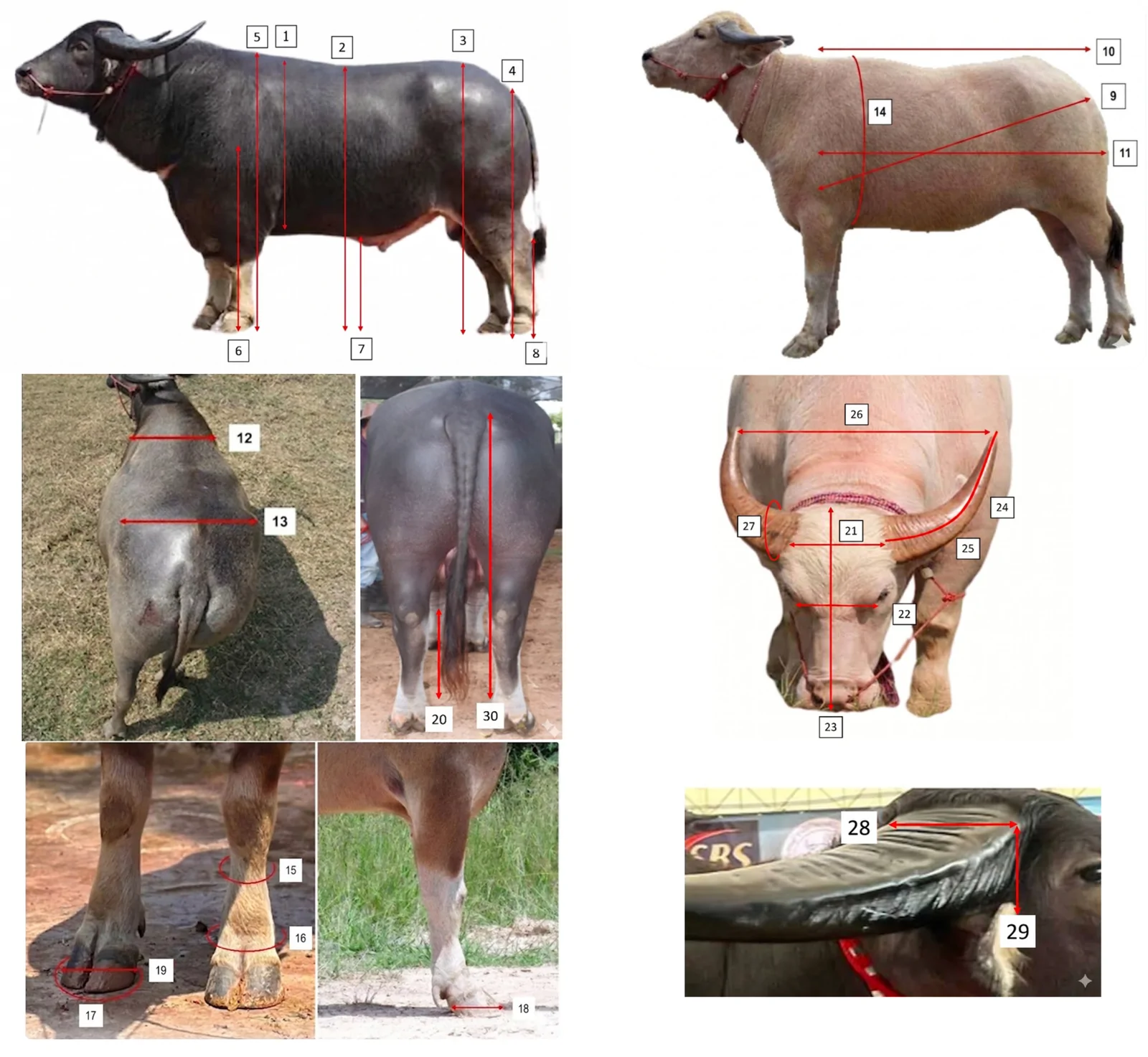
The body measurements as quantitative morphology used in this study were as follows: (1) body depth, (2) back height, (3) hip height, (4) tail head height, (5) shoulder height, (6) point of shoulder height, (7) flank height, (8) rear hock joint height, (9) body length, (10) back length, (11) shoulder length, (12) shoulder width, (13) hip width, (14) heart girth, (15) front cannon circumference, (16) front fetlock circumference, (17) front hoof circumference, (18) front hoof length, (19) front hoof width, (20) tail switch length, (21) forehead width, (22) interocular distance, (23) head length, (24) upper horn length, (25) horn length from base, (26) horn tip distance, (27) horn base circumference, (28) horn base width, (29) horn thickness, (30) tail length.

The characterization of Thai swamp buffalo morphology included qualitative and quantitative data. A qualitative phenotype from 799 animals (666 females and 133 males) includes general physical characteristics, which were as follows: (1) body coat color, (2) horn type, (3) chevron (neck markings), and (4) hair whorls. Quantitative morphology included 30 body measurement, which were as follows: (1) body depth, (2) back height, (3) hip height, (4) tail head height, (5) shoulder height, (6) point of shoulder height, (7) flank height, (8) rear hock joint height, (9) body length, (10) back length, (11) shoulder length, (12) shoulder width, (13) hip width, (14) heart girth, (15) front cannon circumference, (16) front fetlock circumference, (17) front hoof circumference, (18) front hoof length, (19) front hoof width, (20) tail switch length, (21) forehead width, (22) interocular distance, (23) head length, (24) upper horn length, (25) horn length from base, (26) horn tip distance, (27) horn base circumference, (28) horn base width, (29) horn thickness, (30) tail length. Measurements were made with an outside caliper, vernier caliper, tape measure, stadiometer, and level gauge. All measurements followed the Department of Livestock Development (DLD) protocol to reduce bias and keep data collection consistent across locations. The horn types of swamp buffaloes are presented in Table 2, and the body measurement positions are illustrated in Figure 1.

#### 2.2.2. Blood Collection, DNA Isolation, and SNP Data

Blood samples (5–10 mL) from phenotyped buffaloes were collected from the jugular vein into EDTA tubes and stored at 4 °C. Samples were centrifuged at 3000 rpm for 5 min, and 100 µL of the buffy coat was transferred to a microcentrifuge tube. Genomic DNA was extracted using the DNeasy Blood & Tissue Kit (Qiagen GmbH, Hilden, Germany) following the manufacturer’s protocol, including lysis with Buffer AL, ethanol precipitation, column purification, and final elution in 200 µL of elution Buffer.

DNA purity and concentration were assessed using spectrophotometric (Nanodrop, Thermo Fisher Scientific, Wilmington, DE, USA) and fluorometric methods (Qubit Fluorometer, Invitrogen, Thermo Fisher Scientific, Singapore), respectively. Samples meeting the quality criteria for SNP analysis, specifically a concentration of 40–60 ng/µL, an OD_260_/_280_ ratio between 1.80 and 2.00, and an OD_260_/_230_ ratio of greater than 1.50 at a volume of 100 µL, were selected.

To obtain a representative subset for SNP genotyping, animals with complete morphological data were selected using stratified purposive sampling across the seven regions, with proportional representation at the province and local levels. DNA samples that passed laboratory quality control were verified by agarose gel electrophoresis and genotyped with the 90K Axiom^®^ Buffalo SNP Genotyping Array (Thermo Fisher Scientific, Santa Clara, CA, USA). General quality control filtering removed SNPs with call rate < 90%, animals with call rate < 90%, SNPs with minor allele frequency (MAF) < 0.05, SNP showing significant deviation from HWE (*p* < 10^−10^, monomorphic or unmapped markers. The dataset initially contained 123,040 SNPs. After QC filtering and removal of 4891 sex-chromosome SNPs, 21,524 autosomal SNPs remained prior to LD pruning. Linkage disequilibrium (LD) pruning was then applied using an r^2^ threshold of 0.2, and additional 2594 SNPs. Consequently, after all QC steps, 474 animals and 18,930 SNPs (LD-pruned dataset) and 21,524 SNPs (unpruned dataset) were retained. The unpruned dataset was used for ROH analysis, whereas the LD-pruned dataset was used for all other downstream analyses. SNP positions and genomic coordinates provided in the Axiom^®^ Buffalo 90K SNP Genotyping Array are based on the *Bubalus bubalis* reference genome assembly UMD_CASPUR_WB_2.0. The summary of SNP passed for each QC steps is given in Supplement Table S1.

### 2.3. Data Analysis

#### 2.3.1. Descriptive Statistics and Least Square Mean Analysis

Qualitative traits were summarized as counts and percentages within each regional population. Quantitative traits, as illustrated in Figure 1, were analyzed using an ordinary linear mixed model that included fixed effects of region, age, and sex, together with random effects of province and farm. Mixed-model procedures were applied. The statistical model was specified as follows:

$$y_{ijklmn}=\mu +R_{i}{+A}_{j}+S_{k}+{R*S}_{ik}+P_{l}+F_{m(l)}+\varepsilon_{ijklmn}$$

where $y_{ijklmn}\;$*=* quantitative morphological traits; μ = overall mean; $R_{i}\;$= effect of region; $A_{j}$ = effect of age (1–10 years); $S_{k}\;$= effect of sex (male and female); $P_{l}\;$= effect of province; $F_{m(l)}$ = effect of farm nested within province; $\varepsilon_{ijkl}\;$= random error term; all random effects were assumed to follow a normal distribution with mean zero.

Because the interaction between region and sex was significant (*p* < 0.05) in most traits, regional least-squares means were reported separately within each sex. In addition, specific sex comparisons were conducted within each region.

LS-means analyses performed using PROC MIXED and Trait distributions were visualized by PROC SGPLOT in SAS Studio Online version 3.84 (SAS^®^ OnDemand for Academics, SAS Institute Inc., Cary, NC, USA; https://welcome.oda.sas.com accessed on 15 June 2026). Variance components were estimated using restricted maximum likelihood (REML). 

#### 2.3.2. Genetic Characterization and Clustering

Principal Component Analysis was performed separately for morphological and SNP datasets. In the morphology PCA, only non-missing traits of 768 individual buffaloes were treated as observations and 20 quantitative body-measurement traits as variables, with individuals containing missing values removed. For the SNP-based PCA, the input matrix included 374 buffaloes and 18,930 autosomal SNPs retained after quality control, LD pruning, and removal of sex-chromosome markers. For morphology-based PCA, all quantitative traits were centered and scaled to unit variance before analysis because body measurements differed in magnitude and variance. This prevented large-scale traits from dominating the principal components. For SNP-based PCA, genotypes were mean-centered prior to analysis but were not scaled to unit variance. PCA was performed using individual-level genotype data coded as 0, 1, and 2, after mean imputation of missing genotypes. Thus, morphology PCA used standardized phenotypic traits, while SNP PCA used centered allele-dosage data. Eigenvalues, variance explained, and the first two principal components were used to visualize genomic relationships among regional populations.

Canonical Discriminant Analysis was performed to assess differentiation among the seven predefined regional groups. This method maximizes among-group variation relative to within-group variation and identifies canonical variables that best separate populations. Mahalanobis distances from canonical scores were used to quantify regional divergence, while CDA plots and canonical structure coefficients were used to evaluate group separation and identify the main contributing morphological or SNP-derived variables [15].

Hierarchical clustering was performed separately for morphological and SNP allele-frequency datasets. For morphology-based clustering, standardized traits were used to calculate Euclidean distances among regions. For SNP-based clustering, regional allele frequencies were used to calculate identity-by-state (IBS)–derived genetic distances. IBS was defined as the average genotype similarity between pairs of individuals across all SNP loci. UPGMA and Ward’s method were then applied to generate dendrograms [7,16,17,18]. Node support was assessed using 1000 bootstrap replications by resampling body traits for the morphological dataset and SNP markers for the genomic dataset [12,19]. Bootstrap support for taxa-level dendrograms was evaluated using 1000 replicates. Morphological analyses resampled standardized phenotypic traits, whereas SNP analyses resampled SNP loci from the taxa-level allele-frequency matrix, both with replacement. For each replicate, distances and trees were recalculated, and node support was reported as the percentage of replicate trees recovering the original clade.

The PCA and CDA and hierarchical clustering and dendrogram construction together with the bootstrap values were calculated using R script in RStudio version 2025.09.2+418 [20].

#### 2.3.3. Genetic Structure and F-Statistics

The genetic structure was examined at the genome level using the LD-pruned autosomal SNP dataset, which provides an appropriate basis for calculating F-statistics. To assess genetic diversity and population differentiation, the Polymorphic Information Content (PIC) and Wright’s F-statistics (FST, FIS, and FIT) were calculated. These indices provide a robust framework for quantifying heterozygosity and detecting genetic structure within and among populations, as suggested by Nei [16]. The criteria for genetic differentiation and inbreeding were described by Weir and Cockerham [21]. The criteria for pairwise comparisons of F-statistics for genomic data were applied based on recommended approaches for population structure and F-statistics interpretation [22,23,24]. Confidence intervals for global and pairwise Weir and Cockerham F-statistics were estimated by nonparametric bootstrap resampling of SNP loci with replacement. A total of 1000 bootstrap replicates were performed, while individuals and population assignments were kept fixed.

#### 2.3.4. Genomic Structure and Admixture Inference

Population structure and individual ancestry proportions were inferred with sNMF in the LEA R package [25,26] for K = 2–15 with 20 independent runs per K. Final inference should use the LD-pruned autosomal dataset and report replicate-level cross-entropy, mean and variability by K, alignment and stability of ancestry components, missing-genotype treatment, and sensitivity to the ancestry-assignment threshold. K = 3, K = 6, and K = 7 are interpreted as alternative hierarchical resolutions rather than as a single objectively optimal solution.

Q-values from the best run for each K were extracted to estimate each individual’s ancestry proportions and generate admixture plots. For each K, Q-values were obtained from the run with the lowest cross-entropy. The ancestry component with the highest Q-value was identified as the dominant component for each individual and was used, together with geographic region, to order individuals in the admixture plots. No fixed Q-value threshold was applied for discrete ancestry assignment. The full distribution of ancestry proportions was retained to characterize shared ancestry and admixture among regional populations. Bar plots were created in RStudio version 2025.09.2+418 using the base bar-plot function, with individuals ordered by sampling region and dominant ancestry to aid interpretation of genetic structure, admixture, and regional differentiation in Thai swamp buffalo.

#### 2.3.5. Runs of Homozygosity (ROH)

The unpruned SNP data were used to perform ROH. The following criteria were applied: (i) minimum number of SNPs included in a ROH is 15, (ii) minimum ROH length is 1 Mb, (iii) maximum distance between two consecutive SNPs in a ROH is 1 Mb, (iv) no allowance of heterozygous or missing genotype within a ROH, and (v) window size for recurrent ROH and hotspot summary is 1 Mb. Runs of homozygosity (ROH) were identified on autosomes and summarized in the 1–2, 2–4, 4–8, 8–12, 12–16, and >16 Mb classes. A unique ROH was defined as a homozygous segment that starts and ends at the same chromosomal positions. ROH hotspots were defined as SNP level regions where at least 20% of individuals carried an ROH and the SNPs fell within the top 1% of ROH frequency distribution. Recurrent ROH hotspots were defined as 1-Mb windows in which more than 20% of samples carried ROH, and their regional frequencies were visualized using a heatmap.

A fraction of the genome in runs of homozygosity (F_ROH_) was estimated as follows:

$$F_{ROH}=\frac{\sum L_{ROH}}{L_{AUTO}}$$

FROH was calculated as the summed length of qualifying autosomal ROH divided by one explicitly defined autosomal genome length.

Regional means of F_ROH_, ROH number, total ROH length, percent SNPs in ROH, hotspots, and recurrent patterns were summarized to assess genomic autozygosity, recent inbreeding, and subpopulation diversity.

#### 2.3.6. Tools and Software

SAS Code for LS-means analysis with mixed model, and R script for SNP-QC, PCA/CDA, and dendrogram construction with bootstrap, F-statistics, and all pairwise comparisons with bootstrap, Admixture, and cross-entropy analysis and plot, and ROH distribution, FROH, hotspot, and recurrent ROH analysis are available at: http://abagkku.in.th/monchai (accessed on 10 June 2026).

## 3. Results

### 3.1. Morphological Diversity of Thai Swamp Buffalo

#### 3.1.1. Qualitative Morphological Variation

Buffalo coat color is mainly determined by skin pigmentation and hair color. In Thai swamp buffaloes, black coats are more common than white. Calves are usually born with long, dense light gray or yellow hair, much of which is lost with age. In this study, black buffaloes had nearly all-black skin (99.57%) with black or dark gray hair, whereas white buffaloes had pink skin (0.43%) with small dark gray or brown freckles, mainly along the back and sides and less often on the underside. Older white buffaloes tended to show more visible subcutaneous freckles.

The horn shape of native Thai swamp buffalo varies by region. The most common types were bowl-shaped (29.46%), crescent (25.19%), and stacked horn (27.13%). Less common horn types included mild-spread horns (8.91%) and wide-spread horns (6.59%), whereas droopy and blunt horns were rare (2.71%), and horn deformities were extremely rare (less than 1%). The bowl-shaped type is found mostly in N1 (13.95%) and N2 (12.41%). The crescent type is found mostly in N1 (17.06%) and is found less than 5% in other regions.

Chevron markings also showed variations. Most buffaloes had two chevrons (53.49%) or one chevron (26.36%). Fewer animals had none (18.99%), three (0.78%), or four (0.39%). These markings usually appear on the neck, chest, and extend toward the brisket. They are typically white and form V- or U-shaped patterns. Regional differences were observed. In the upper (N1) and lower (N2) northern regions, two chevrons were most frequent, and some animals with three or four chevrons were found in the Nam Wa strain in Nan Province. In contrast, buffaloes in the southern (S) region mostly had one chevron (44.4%) or none (42.2%), and no individuals had more than two.

Hair whorl patterns also varied across regions. Some buffaloes had no whorls, whereas others had multiple whorls. The most common pattern was four to five whorls (20.24%). Approximately 13.1% of the animals had no whorls, and a very small number showed as many as 10 (0.40%). This variation reflects the diversity of external traits in the Thai swamp buffalo population.

#### 3.1.2. Quantitative Morphological Variation

LS-means of morphological traits for each region are shown in Table 3. Although the region × sex interaction was significant, overall regional LS-means are presented for descriptive purposes. The least-square means in Table 3 showed regional variation in body conformation among Thai swamp buffaloes. The LS-means, adjusting for age, province, and farm effects, showed clear regional differences in economically important body-conformation traits of Thai swamp buffaloes (Table 3). Overall body size was generally greatest in the Central (C) and NE1 populations, as follows: female/male body length reached 145.5/158.3 cm in C, while back height was 131.8/141.5 cm in C and 133.2/137.0 cm in NE1. In contrast, S2 buffaloes tended to have smaller skeletal dimensions, including back height (116.3/116.0 cm), hip height (119.4/120.1 cm), and shoulder height (119.3/120.6 cm). Regional differences were also evident for body conformation, cannon circumference, and hoof dimensions, traits relevant to overall body capacity, structural soundness, working ability, and indirect assessment of body weight. Significant region × sex interactions found in most traits, indicated that sexual dimorphism differs among regions, so averaged regional comparisons are not meaningful because values depend on sex differences. For example, body length differed markedly between females and males in C (145.5 vs. 158.3 cm), NE2 (129.3 vs. 152.3 cm), and S2 (132.1 vs. 139.6 cm), whereas the sex difference was much smaller in NE1 (145.8 vs. 144.0 cm). Similarly, males exceeded females in back height in C, N1, N2, NE1, and NE2, but not clearly in S1 or S2. Such sex-dependent regional variation is consistent with reported sexual dimorphism in swamp buffalo and indicates that sex-specific regional standards should be considered when using morphometric traits for phenotypic characterization and selection.

### 3.2. Genetics Characterization and Clustering

#### 3.2.1. Principal Component Analysis (PCA)

**Figure 2:**
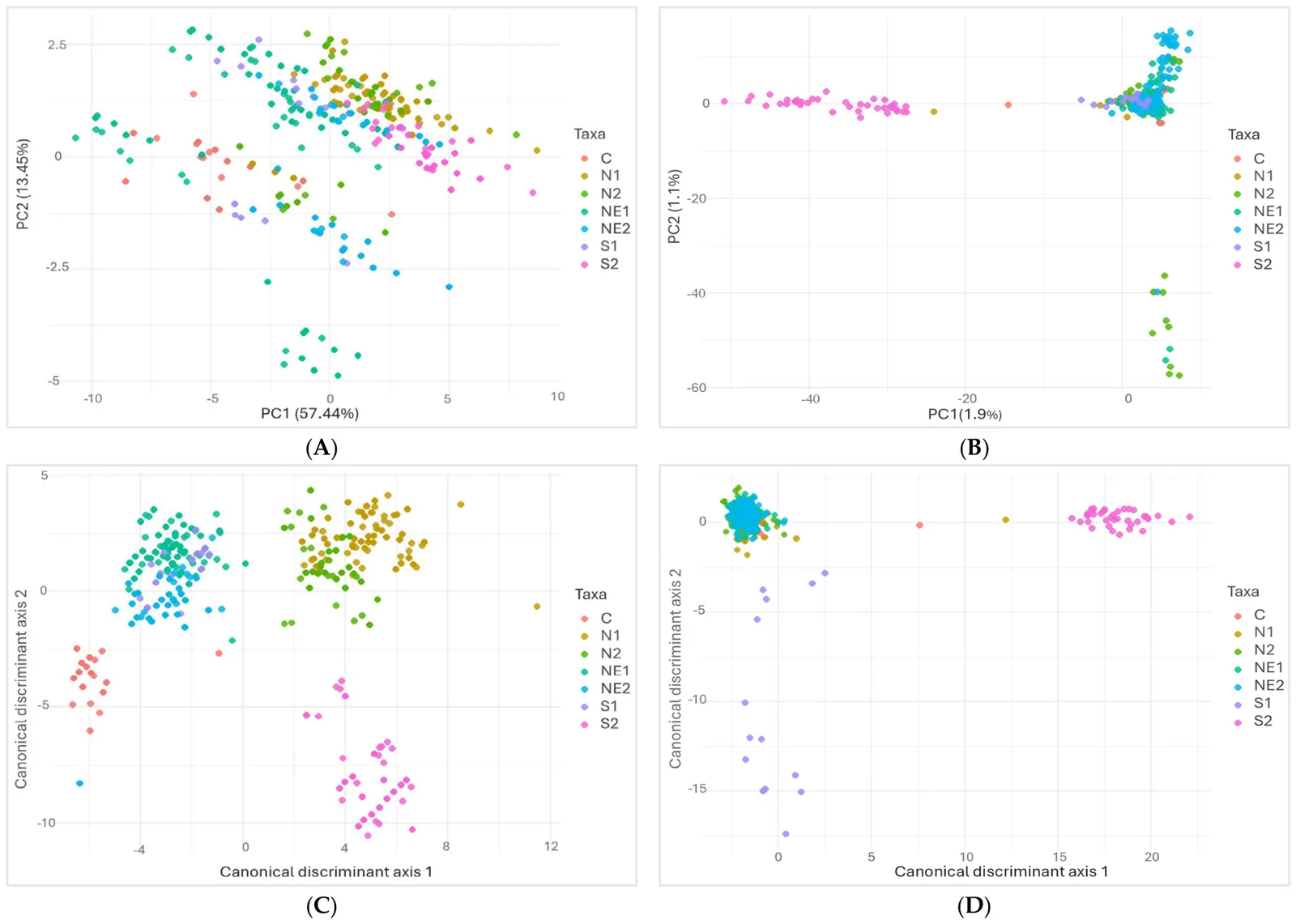
Principal component analysis of standardized morphological traits adjusted for effects of age, sex, province, and farms (**A**) and genome-wide SNP data (**B**), and canonical discriminant plots (**C**,**D**). Points represent individual animals, and colors denote regional populations (C, N1, N2, NE1, NE2, S1, S2).

PCA was used to examine genetic relationships without predefined group assignment. A total of 31 animals were excluded due to missing phenotypes, leaving 768 animals with complete phenotypes for the PCA/CDA analyses. The first two principal components derived from the 30 body conformation traits explained 60.69% (49.18% for PC1 and 11.51% for PC2) of the total phenotypic variation. The first two components based on LD-pruned autosomal SNP explained 3.00% (1.91% for PC1 and 1.1% for PC2) of the total genetic variation. In this study, PCA showed different patterns between morphological and genome-wide SNP data. Morphology-based PCA showed broad overlap among all regions, indicating limited phenotypic separation, although some regional variation was observed (Figure 2A). In contrast, SNP-based PCA (Figure 2B) showed a more structured pattern. S2 was clearly separated toward the negative side of PC1, suggesting distinct genomic differentiation of the Lower South population. Some N2 and NE1 individuals also dispersed along PC2, indicating some within-region genomic variation. Most individuals from the other regions are clustered closely on the positive side of PC1, indicating a possibly strong sharing of genetic background.

#### 3.2.2. Canonical Discriminant Analysis (CDA)

Morphological CDA was used descriptively to examine separation among predefined seven regional populations. The CDA plot provided clearer separation among regional groups, particularly between C, NE1, and NE2. However, substantial overlap remained, suggesting that body conformation traits can distinguish some regional patterns but are insufficient for complete population separation. CDA based on genome-wide SNPs (Figure 2D) showed much clearer regional separation than CDA based on morphology (Figure 2C). The SNP-based CDA separated S2 clearly along canonical discriminant axis 1 and S1 showed greater dispersion along axis 2, suggesting the most within-region variation or sampling effects. Most C, N1, N2, NE1, and NE2 individuals clustered closely together, indicating a possible shared genetic background. Compared with the SNP-based PCA, SNP-based CDA provided stronger discrimination, and confirmed the distinct genomic position of S2 more clearly. Both SNP-based PCA and CDA also support the close genetic relationship among the central, northern, and northeastern populations.

#### 3.2.3. Distance-Based Dendrograms

**Figure 3:**
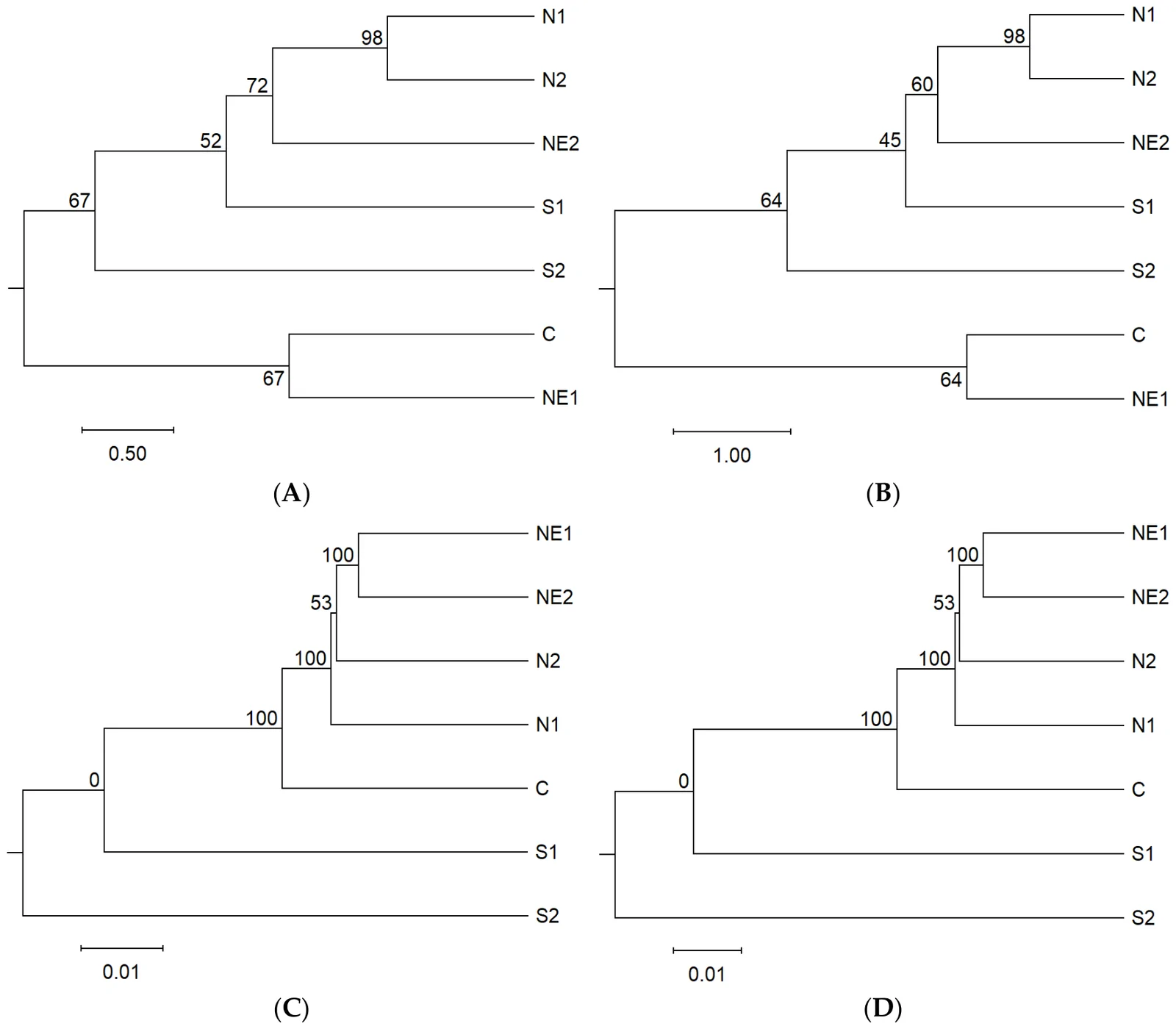
Dendrograms from cluster analyses of standardized body-measurement traits adjusted effects of age, sex, province, and farms using Euclidean distance with (**A**) UPGMA and (**B**) Ward’s method, and genome-wide SNP data using IBS distance with (**C**) UPGMA and (**D**) Ward’s method. Bootstrap support values from 1000 resampling are shown at internal nodes. C = Central, N1 = Upper North, N2 = Lower North, NE1 = Upper Northeast, NE2 = Lower Northeast, S1 = Upper South, S2 = Lower South.

Cluster analysis of the 30 standardized body-measurement traits separated the seven regions into several groups, with some differences between the UPGMA and Ward’s methods. In the morphology-based UPGMA dendrogram (Figure 3A), N1 and N2 formed the closest and most stable cluster (94%), with NE2 joining this northern group at moderate support (70%). S1 and S2 also clustered together but with weak support (39%). In contrast, C and NE1 formed a strongly supported cluster (99%). The morphology-based Ward’s dendrogram (Figure 3B) showed a similar pattern, again supporting the N1–N2 cluster (89), grouping NE2 with the northern cluster (70%), clustering S1 with S2 (61%), and confirming the strong C–NE1 grouping (98%). These results indicate that N1–N2 and C–NE1 were consistent morphological clusters, while S1–S2 was less stable.

SNP-based clustering provided clearer and more consistent genetic relationships. In the SNP-based UPGMA dendrogram (Figure 3C), NE1 and NE2 formed the closest cluster with full support (100%), followed by N1 and N2, indicating strong genomic connectivity among northeastern and northern populations. The Central population fell outside this cluster, while S1 and especially S2 were clearly separated from the other regions. The SNP-based Ward’s dendrogram (Figure 3D) showed the same topology. S2 showed the longest branch distance, confirming it as the most genetically distinct population.

#### 3.2.4. Genetic Structure and F-Statistics

Genome-wide SNP analysis indicated low-to-moderate genetic diversity across the seven Thai swamp buffalo regions (Table 4). The average observed heterozygosity was slightly lower than the expected heterozygosity (H_O_ = 0.323; H_E_ = 0.334), suggesting a mild heterozygote deficiency. Genetic diversity was relatively similar across most regions, with H_O_ ranging from 0.313 to 0.335, whereas the Lower South exhibited the lowest H_O_ (0.285) and H_E_ (0.296). PIC values ranged from 0.238 to 0.270, indicating moderate marker informativeness, with the highest value observed in the Upper Northeast. The average F_ST_ was 0.0170, suggesting generally low genetic differentiation among regions, although higher differentiation was detected in the S2 (F_ST_ = 0.0536) and S1 (F_ST_ = 0.0264). Overall inbreeding was limited, with mean F_IS_ and F_IT_ values of 0.0220 and 0.0386, respectively. The N1 showed the highest positive F_IS_ (0.0453), whereas the S1 had a negative F_IS_ (−0.0935), indicating an excess of heterozygosity relative to Hardy–Weinberg expectations within this regional population.

Pairwise F-statistics showed mostly low but detectable genetic differentiation among the seven Thai swamp buffalo regions (Table 5). Most FST values among C, N1, N2, NE1, and NE2 were very low (0.004–0.009), indicating close genomic relationships among these populations. Higher differentiation was observed in comparisons involving southern populations, especially C–S2 (0.061), N1–S2 (0.056), N2–S2 (0.058), and S1–S2 (0.080), suggesting clearer regional subdivision in the South. Pairwise (FIS) values were generally low or near zero, with some negative values in S1, indicating minimal heterozygote deficiency and little evidence of inbreeding. Pairwise FIT values were also low, although higher values involving S2, particularly N1–S2 (0.104) and C–S2 (0.100), suggested greater total-population differentiation in these comparisons. The comparison result showed that S2 exhibited the clearest genomic distinction.

Bootstrap confidence intervals for global and pairwise F-statistic values are provided in Supplement Table S2 and support the same pattern of low differentiation among mainland populations and elevated differentiation involving the Lower South.

#### 3.2.5. Genomic Structure and Admixture Inference

**Figure 4:**
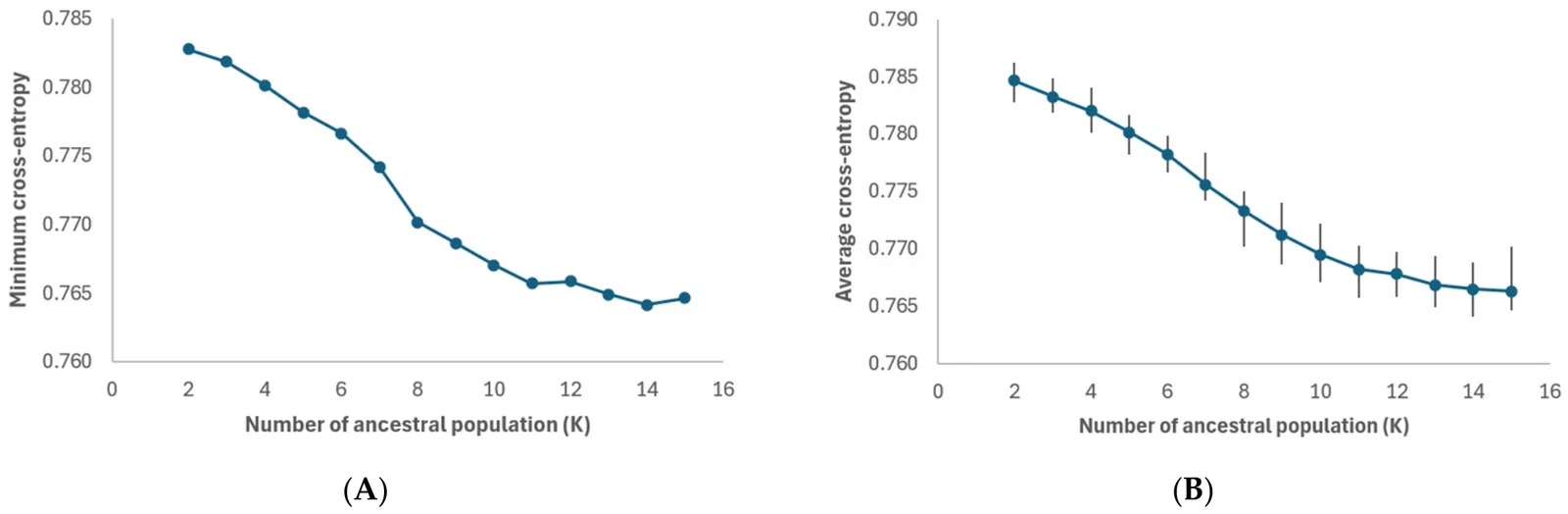
Cross-entropy profiles from genome-wide SNP-based sNMF ancestry analysis across different numbers of ancestral populations (K = 2–15). (**A**) Minimum cross-entropy values from the best replicate run for each K, (**B**) Average cross-entropy values across 20 replicate runs for each K, with error bars indicating range among runs.

Cross-entropy in Figure 4 decreased as K increased and reached its minimum at K = 14, but the curve plateaued after K ≈ 10, indicating that higher K values might capture individual variation rather than regional structure. However, the entropy decreased most rapidly before K = 8. At K = 8, the ancestry patterns were clear and biologically interpretable, resolving major regional structure without the fragmentation seen at higher K values. Therefore, K = 8 was considered the most appropriate representation of regional structure in Thai swamp buffaloes. Cross-entropy for all runs and K also provided in Supplement Table S3.

**Figure 5:**
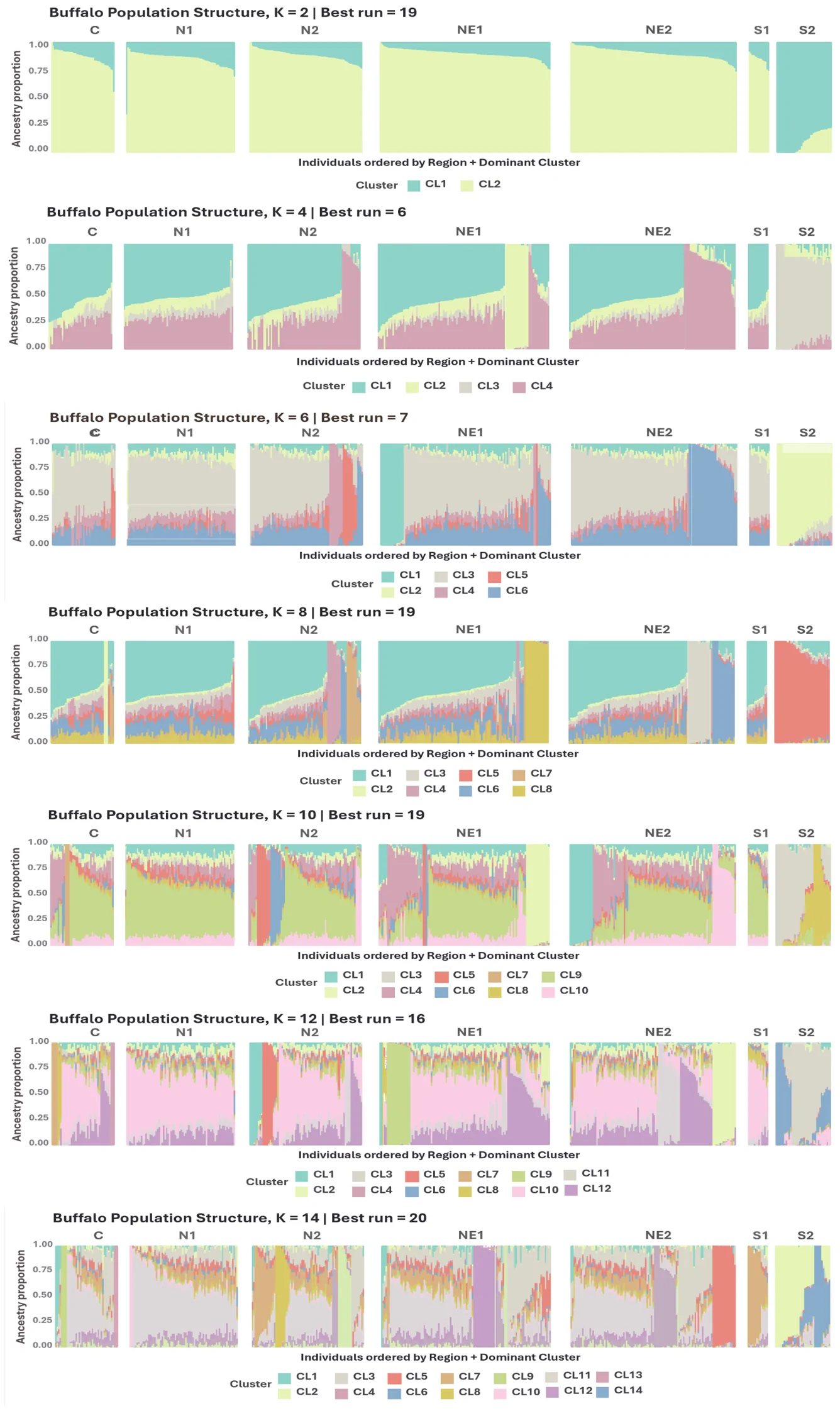
Genome-wide SNP-based admixture structure of Thai swamp buffalo populations inferred from the best sNMF run for each K value from K = 2 to K = 15. Each vertical bar represents an individual, and colors indicate the inferred individuals grouped by region and ordered by the dominant ancestry cluster within each region. Sample numbers were C = 43, N1 = 74, N2 = 76, NE1 = 116, NE2 = 113, S1 = 14, and S2 = 38. C = Central, N1 = Upper North, N2 = Lower North, NE1 = Upper Northeast, NE2 = Lower Northeast, S1 = Upper South, and S2 = Lower South.

From admixture plot in Figure 5, Biologically, K = 2 reflected the main north and south contrast, while K = 4–6 revealed the core regional patterns. Central, northern, northeastern, and Upper South populations shared similar ancestry profiles, whereas the Lower South was consistently the most distinct. At K = 8, the C, N, and NE regions showed similar ancestry profiles and high within-region admixture, reflecting shared ancestry and ongoing gene flow. The S1 appeared as an intermediate group with mixed ancestry from both the mainland and southern differentiations. In contrast, the S2 was the most genetically distinct, dominated by a single ancestry cluster and exhibiting the lowest diversity, consistent with its higher genetic differentiation and FST values relative to other regions.

Admixture results were consistent with the SNP-based PCA, which showed a geographic gradient rather than complete regional separation. The distinct position of the S2 and the intermediate placement of the S1 matched their ancestry profiles. Dendrograms grouped C, N, and NE populations closely and placed the southern populations, especially the S2, on more divergent branches.

#### 3.2.6. Runs of Homozygosity (ROH)

ROH analysis from unpruned SNP data characterized the genomic autozygosity patterns across the Thai swamp buffalo population. Across all regions, 14,703 ROH segments were detected, with an average of 31.0 ROH per animals, ranging from 25.9 to 59.6 segments per animal. The average length of ROH varied among regions from 52.1 to 179.9 Mb, with FROH values of 0.026–0.089 (Table 6).

In addition, ROH length profiles indicated clear population differentiation. Most regions shared similar ROH metrics, reflecting low differentiation and extensive historical gene flow. The Upper South showed a transitional pattern, while the Lower South was the most differentiated, with elevated homozygosity and long ROH segments indicating reduced connectivity and a smaller effective population size. The F_ROH_ values further supported these regional differences. Lower South had the highest F_ROH_ (0.089), suggesting the greatest fraction of the autosomal genome in homozygous segments, whereas Upper South had the lowest F_ROH_ (0.026). Most ROH segments were short, mainly 1–2 Mb (7485 segments) and 2–4 Mb (5471 segments), indicating that historical relatedness and older shared ancestry contributed substantially to the ROH landscape. Longer ROH segments were less frequent, with 304 segments in 8–12 Mb, 96 in 12–16 Mb, and 56 in greater than 16 Mb, suggesting limited but detectable recent inbreeding in some regions. ROH segments distribution by region is given in Supplement Figure S1.

**Figure 6:**
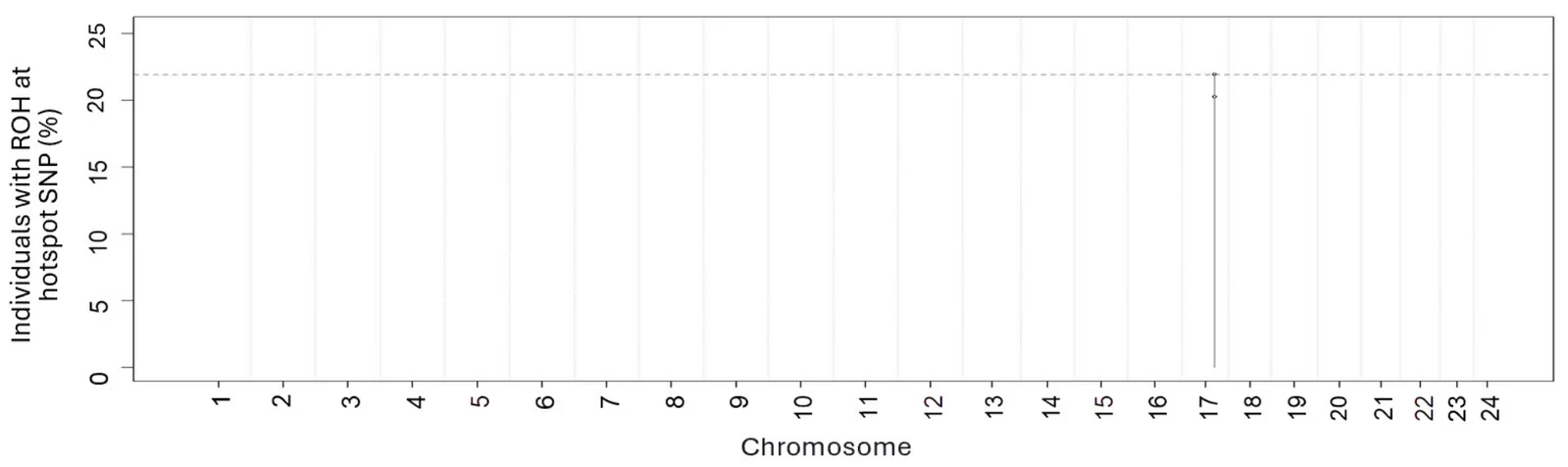
Genome-wide ROH hotspot SNPs across Thai swamp buffalo. SNP-level ROH hotspots were defined as loci located within ROH segments in at least 20% of all individuals; the dashed line indicates the threshold.

The SNP-level ROH hotspot analysis showed that only a few hotspot SNPs were shared across all samples, with clear signals mainly on chromosomes 17 (Figure 6), and the strongest peak on chromosome 17. This indicates that only limited autozygous regions are common across the entire population. Regional hotspot patterns were highly heterogeneous (Supplement Figure S2). The Lower South (S2) showed the strongest and most widespread hotspots across many chromosomes, while NE1 and NE2 showed few hotspots.

**Figure 7:**
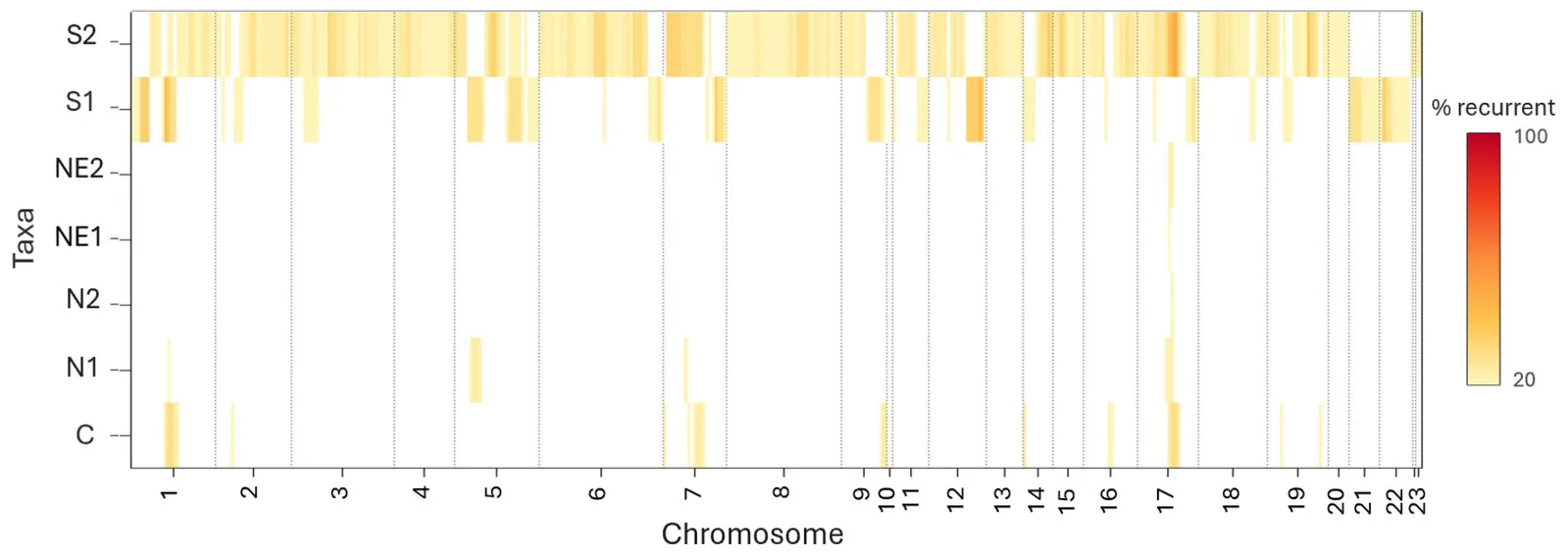
Regional heatmap of recurrent runs of homozygosity (ROH) hotspot windows detected in more than 20% of individuals within each region. Rows represent regional populations, and columns represent genomic windows ordered by chromosome number and physical position. Chromosome boundaries and genomic coordinates are shown along the x-axis to indicate the location of recurrent ROH regions across the buffalo genome. C = Central, N1 = Upper North, N2 = Lower North, NE1 = Upper Northeast, NE2 = Lower Northeast, S1 = Upper South, and S2 = Lower South.

The filtered recurrent ROH window heatmap (Figure 7) provided a clearer population-level view. S2 showed the most extensive recurrent ROH windows across many chromosomes, indicating strong autozygosity and shared homozygous segments within the region. S1 displayed several recurrent windows but with less continuity, suggesting localized shared ancestry. Central showed only a few recurrent windows, and N1, N2, NE1, and NE2 exhibited very limited high-recurrence windows under the stricter threshold, reflecting lower population-level recurrence or greater heterogeneity. The unfiltered heatmap recurrence across all regions also showed in Supplement Figure S3; similar signals were strongest in S2 and, to a lesser extent, S1 and Central.

## 4. Discussion

Integration of phenotypic and genomic evidence indicates that regional diversity in Thai swamp buffalo reflects both inherited differentiation and environmentally mediated variation. Central buffaloes had comparatively large body frames, whereas southern animals, particularly S1, were smaller. Although these traits were adjusted for age, sex, and location, the phenotypic differences did not correspond to comparable separation in the unsupervised genomic analyses. Body dimensions are still responsive to nutrition, production systems, management, and farmer selection. Therefore, phenotypic similarity does not necessarily indicate close ancestry, and morphological divergence does not necessarily represent genome-wide isolation [4]. The morphological associations between N1–N2 and C–NE1 may consequently reflect comparable production environments or selection for similar functional body types rather than identical demographic histories. Comparable local morphological differentiation has been reported between Thai dwarf and conventional swamp buffaloes and between Kalang buffaloes in Indonesia and Thale Noi buffaloes in southern Thailand [27,28,29]. Smaller body dimensions in southern populations may also be associated with humid lowland environments and coastal adapted production systems, consistent with phenotypic variation reported in Indonesian and Philippine swamp buffaloes [18,28]. Nevertheless, broad morphological overlap among most Thai regions reinforces the need to interpret body measurements together with genomic evidence.

Genomic analyses identified a largely connected gene pool comprising C, N1, N2, NE1, and NE2. Their low pairwise differentiation, overlapping ancestry profiles, and limited recurrent ROH support extensive shared ancestry and historical animal exchange. Traditional livestock movement, communal grazing, and farmer-mediated exchange among agricultural regions may have maintained gene flow and reduced divergence, particularly in northern and northeastern Thailand [2]. This interpretation agrees with genome-wide evidence that buffalo structure has been shaped by post-domestication migration and admixture rather than geographic distance alone [7]. Mitochondrial analyses likewise indicate historical expansion, dispersal, and subsequent regional restructuring of Southeast Asian swamp buffalo during domestication [3]. Heterogeneity within N2, NE1, and NE2 may therefore represent local herds, founder differentiations, or breeding networks embedded within a broadly connected mainland population rather than deeply differentiated regional breeds.

The southern populations showed the clearest geographic structure. S1 occupied an intermediate genomic position and carried ancestry components shared with main population (C, N1, N2, NE1, NE2) and S2 buffaloes, suggesting a transitional or contact population along the peninsula. This pattern resembles ancestry gradients associated with historical migration and crossbreeding in Asian buffalo [7,30]. Interpretation of S1 nevertheless requires caution because its small sample size may inflate ancestry dispersion and reduce the precision of diversity estimates. S2 was consistently differentiated across PCA, ancestry, genetic-distance, F-statistic, and ROH analyses, supporting its recognition as a distinct Lower South differentiation. This differentiation is likely driven by geographic separation along the Thai–Malay Peninsula, limited breeding exchange, small local herd sizes, founder effects, and genetic drift. Comparable isolation-related divergence has been reported in Asian swamp buffalo populations [8], and genomic studies in Chinese buffalo similarly show that population size, linkage disequilibrium, and breeding history strongly shape regional genetic structure [12].

ROH analysis provides demographic insight beyond admixture and heterozygosity. The predominance of short ROH across most regions suggests ancient relatedness, historical population contraction, or background autozygosity rather than widespread recent inbreeding, consistent with comparative studies in river and swamp buffalo [30]. In S2, reduced heterozygosity together with high FROH, longer ROH segments, and extensive recurrent windows indicates accumulated autozygosity and a smaller effective breeding population. Similar associations between long ROH, restricted sire use, demographic contraction, and inbreeding have been documented in Italian Mediterranean, Brazilian, and Iranian river buffaloes [31,32,33]. These patterns support conserving S2 as a distinct regional differentiation while improving mating management to increase effective population size and limit further autozygosity without eroding its genomic identity.

The markedly small sample size in S1 may have reduced statistical power in both the morphometric and genomic analyses and may have limited the accurate estimation of genetic diversity. Therefore, regional findings for S1 should be interpreted with caution and considered preliminary until they can be validated using a larger and more representative sample.

## 5. Conclusions

This study highlights meaningful regional differentiation within Thai swamp buffaloes, especially the distinctiveness of the Lower South differentiation and the cohesive genetic background shared among Central, northern, and northeastern populations. These patterns emphasize how geography, herd structure, and long-standing management practices shape regional diversity. Recognizing the Lower South as a distinct differentiation contributes meaningful context to global buffalo research, where most studies remain limited in sample size and geographic coverage. Insights from Thailand therefore contribute important comparative evidence for understanding how isolation, admixture, and local adaptation influence swamp buffalo diversity across Asia.

Given the limited number of genomic studies on swamp buffalo worldwide, these findings offer additional context that can support future work on demographic history, conservation genetics, and regional adaptation. However, caution is needed when generalizing results, as local management, sampling imbalance, and environmental differences can influence both phenotypic and genomic patterns. For academic applications, regional context should be explicitly considered when interpreting genetic structure, designing conservation programs, or comparing Thai buffaloes with other populations. Increasing sample sizes in underrepresented regions will further improve the reliability of future genomic structure analyses.

## Figures and Tables

**Table 1:** Geographic distribution and sampling structure of Thai swamp buffalo populations.

Region	Provinces (N Sample)	Coordinate Range (Approx.)	Ecotype and Local Specificity
Central (C, N = 62)	Nakhon Nayok (30) Chanthaburi (32)	Lat: 14°12′ N Long: 101°11′ E	Ecotype: Central plains buffaloes; highly tolerant of wetlands and seasonal flooding. Local Specificity: Traditionally used for heavy labor in rice paddies; generally larger in body size due to selective breeding for power.
Upper Northern (N1, N = 172)	Nan (63), Lampang (17), Lamphun (30), Phayao (62)	Lat: 18°20′–19°23′ N Long: 98°52′–101°08′ E	Ecotype: Mountainous and valley buffaloes; hardy in cold weather and steep terrain. Local Specificity: Raised in free-roaming forest systems; isolated by high mountain ranges.
Lower Northern (N2, N = 107)	Phitsanulok (30), Sukhothai (29), Uttaradit (48)	Lat: 16°46′–17°34′ N Long: 99°39′–100°54′ E	Ecotype: River basin buffaloes (Nan and Yom Rivers). Local Specificity: A transition zone between the Northern and Central styles with characteristics that are a blend of highland and lowland populations.
Upper Northeastern (NE1, N = 189)	Khon Kaen (40), Nakhon Phanom (82), Roi Et (36) Mahasarakham (31)	Lat: 16°03′21.1″–17°37′48.5″ N Long: 102°22′53.4″–104°31′06.4″ E	Ecotype: Khorat Plateau–Mekong River Basin swamp buffaloes Local Specificity: Adapted to dry seasonal zones with long-distance grazing, rice-based smallholder systems, and use of public grazing lands, post-harvest rice fields, and seasonally flooded forests (Bung Tam). This region functions as one of Thailand’s main buffalo production centers.
Lower Northeastern (NE2, N = 171)	Surin (120), Amnat Charoen (16), Yasothon (15), Ubon Ratchathani (20)	Lat: 14°52′24.8″–16°02′22.1″ N Long: 103°06′35.8″–104°50′18.6″ E	Ecotype: Lower Mun–Chi River Basin and southern Korat Plateau swamp buffaloes Local Specificity: Adapted to hot-dry lowlands, paddy-field grazing, seasonal wetlands, floodplains. Strong cultural linkage with buffalo raising and retain locally adapted morphology by traditional selection.
Upper Southern (S1, N = 38)	Krabi (38),	Lat: 7°42′–8°24′ N Long: 99°04′–99°58′ E	Ecotype: Flat and low-lying inland wetlands. Local Specificity: Adapted to the “8 months rain, 4 months sun” tropical monsoon climate; typically have thinner coats to handle high humidity.
Lower Southern (S2, N = 60)	Trang (8), Satun (20) Nakhon Si Thammarat (32)	Lat: 6°59′–7°05′ N Long: 99°36′–99°40′ E	Ecotype: Area along the Andaman coast inhabit coastal plains and island environments. Local Specificity: Often integrated into mixed-farming systems, such as rubber plantations; seagrass is one of the local feed resources.

**Table 2:** Horn types of Thai swamp buffalo.

Horn Type	Description	Horn Type	Description
Mild spread 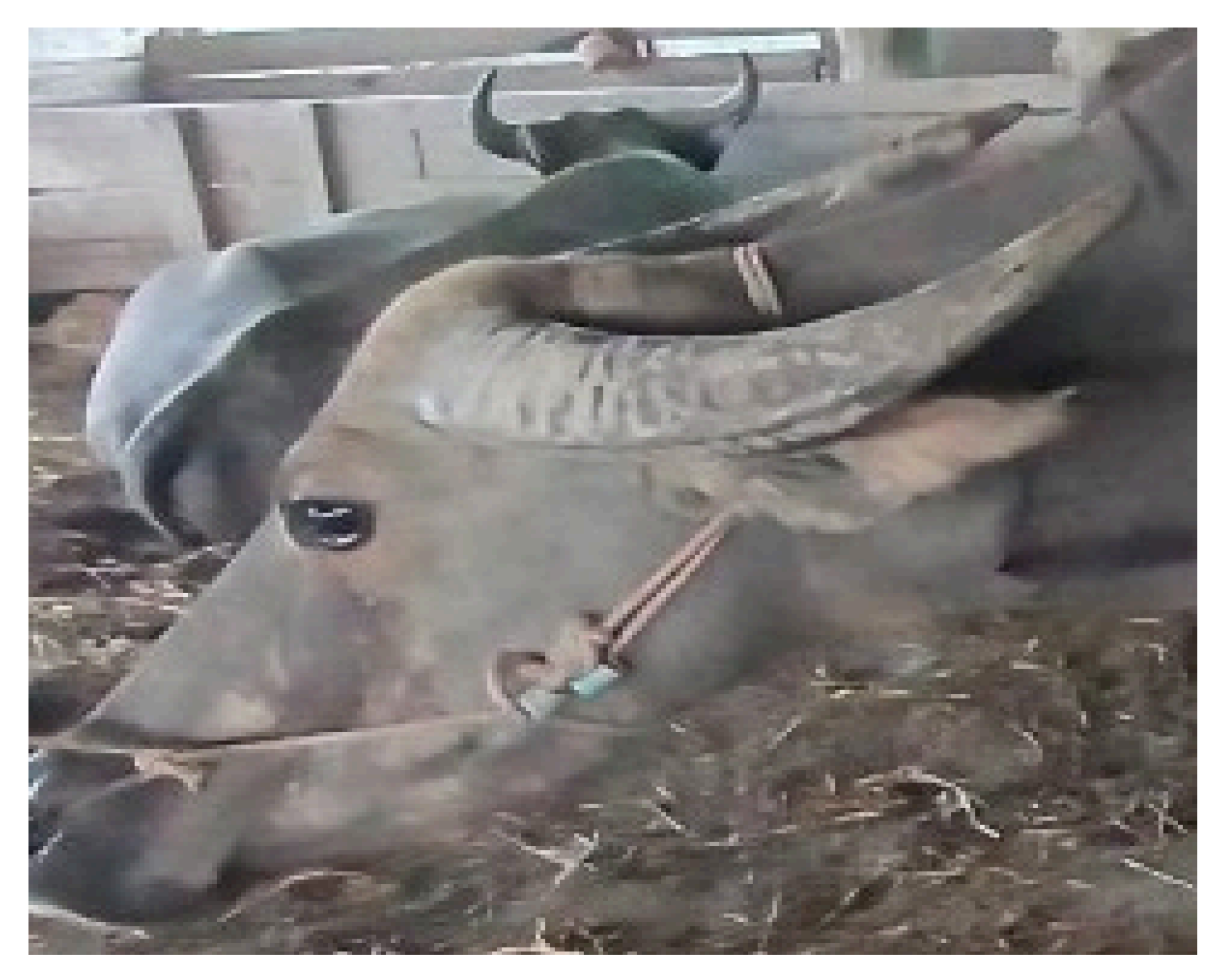	This horn type extends laterally with little upward curvature and is not too long. The horns sweep outward and slightly backward, tapering from a thick base to form a compact structure suited to dense or confined habitats.	Bowl-like 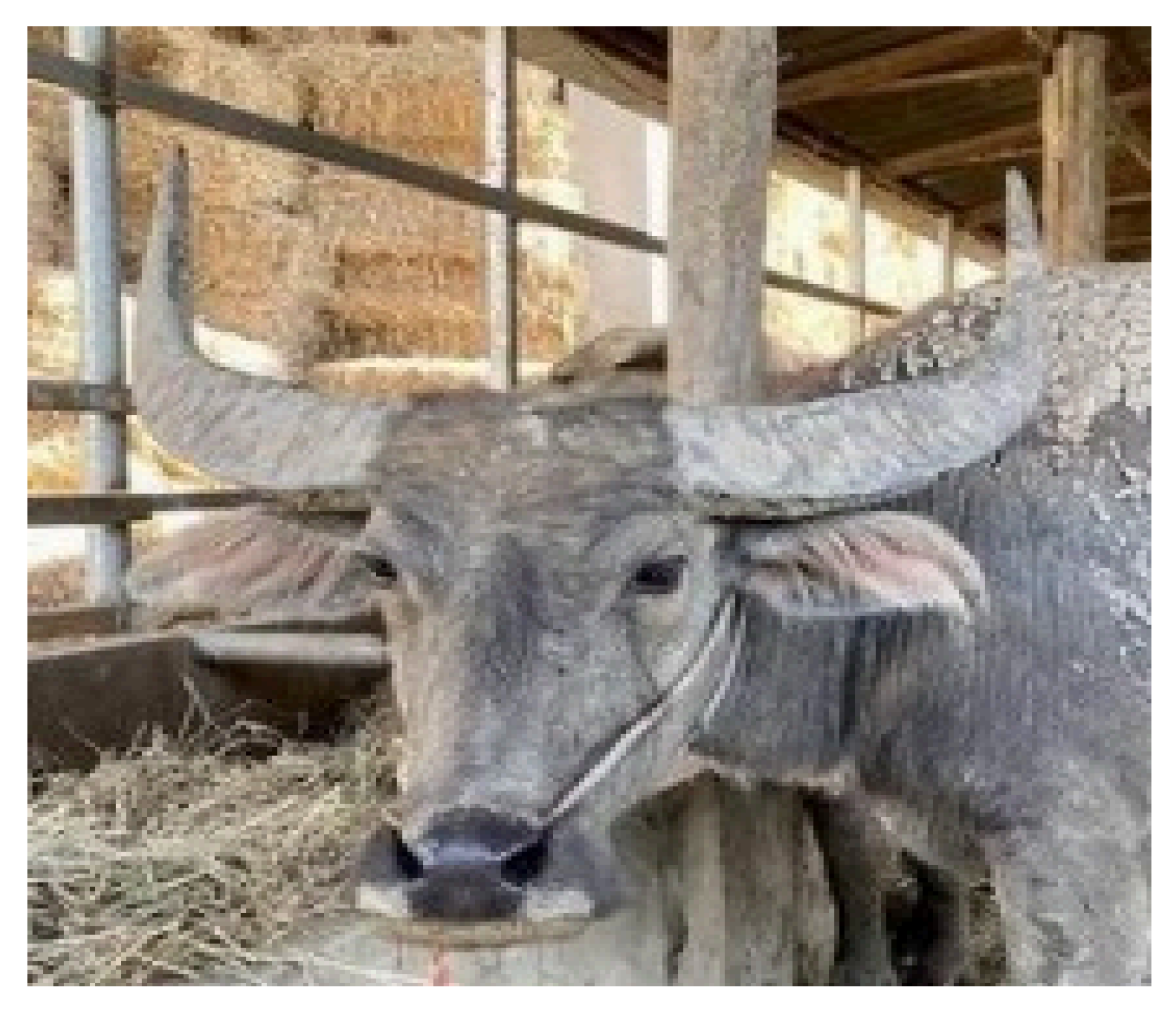	This horn type curves upward and inward in a smooth, bowl-like arc, with tips nearly meeting above the head. Its distinctive, cupped shape is traditionally valued.
Crescent 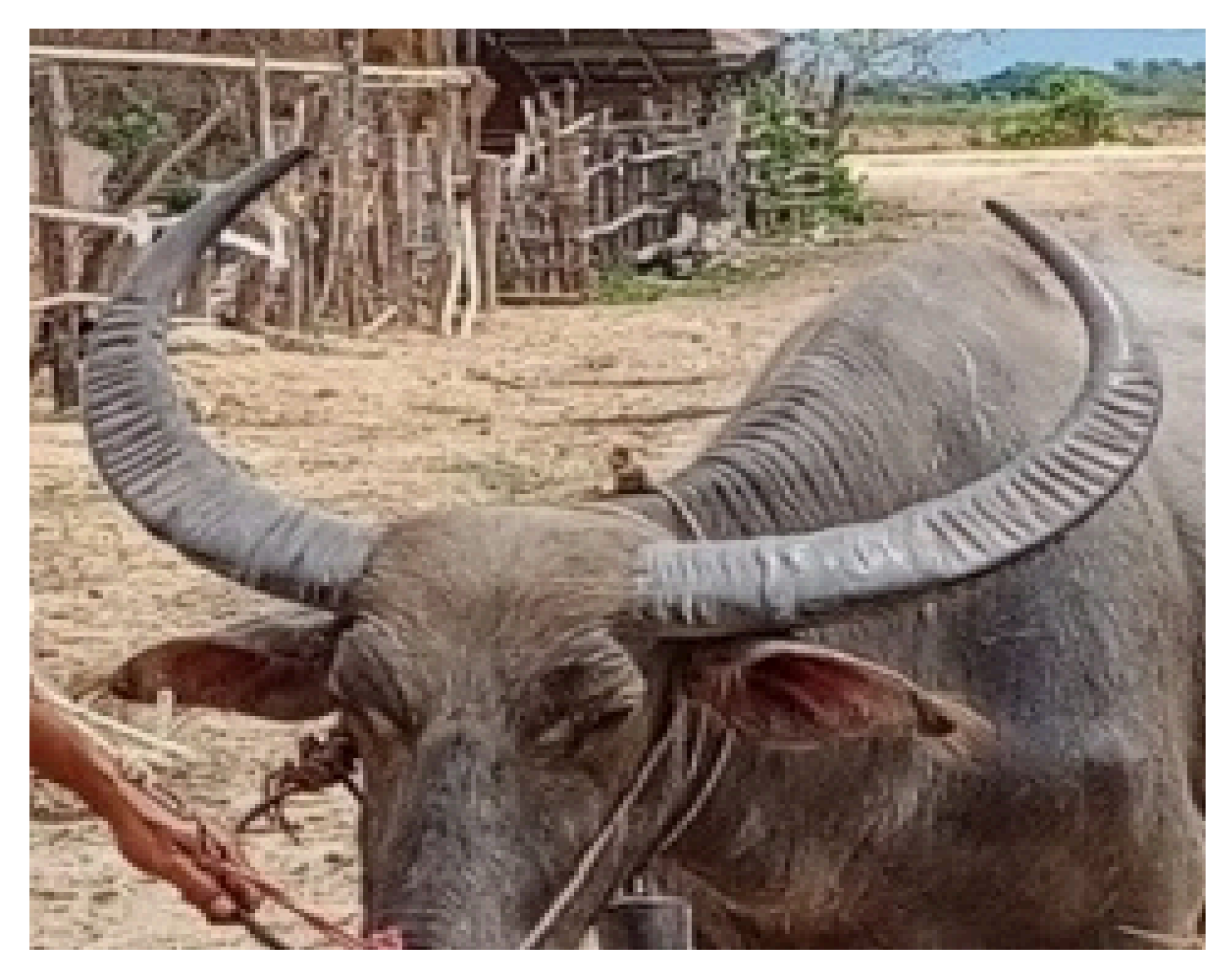	This horn type forms a smooth semicircular curve, sweeping outward, then upward and inward. Its balanced arc enhances the visual display.	Droopy 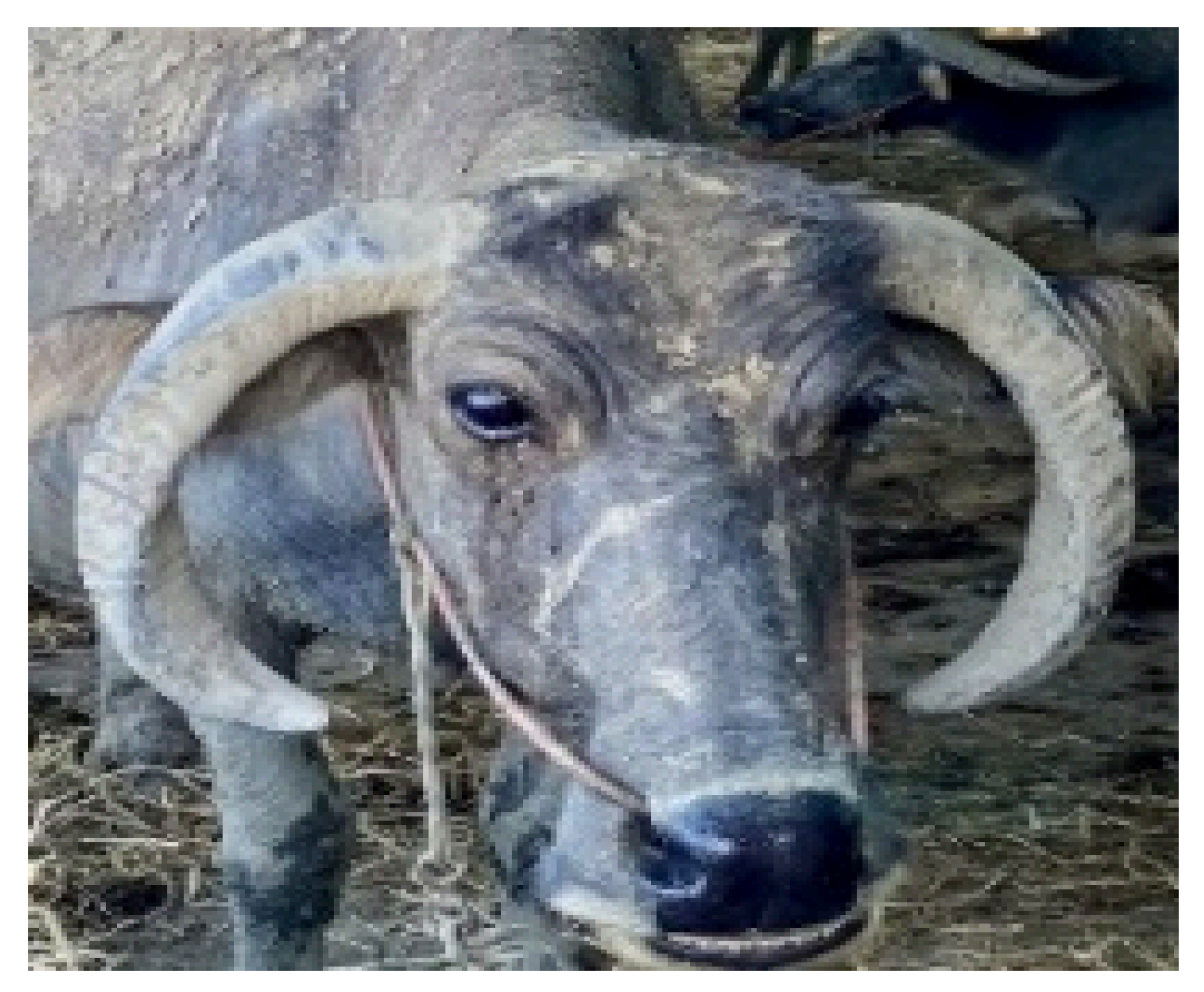	Droopy horns curve downward from the lateral skull base and then inward; the tips converge toward the face, forming a loop-like or hanging semicircular shape when viewed frontally.
Wide spread 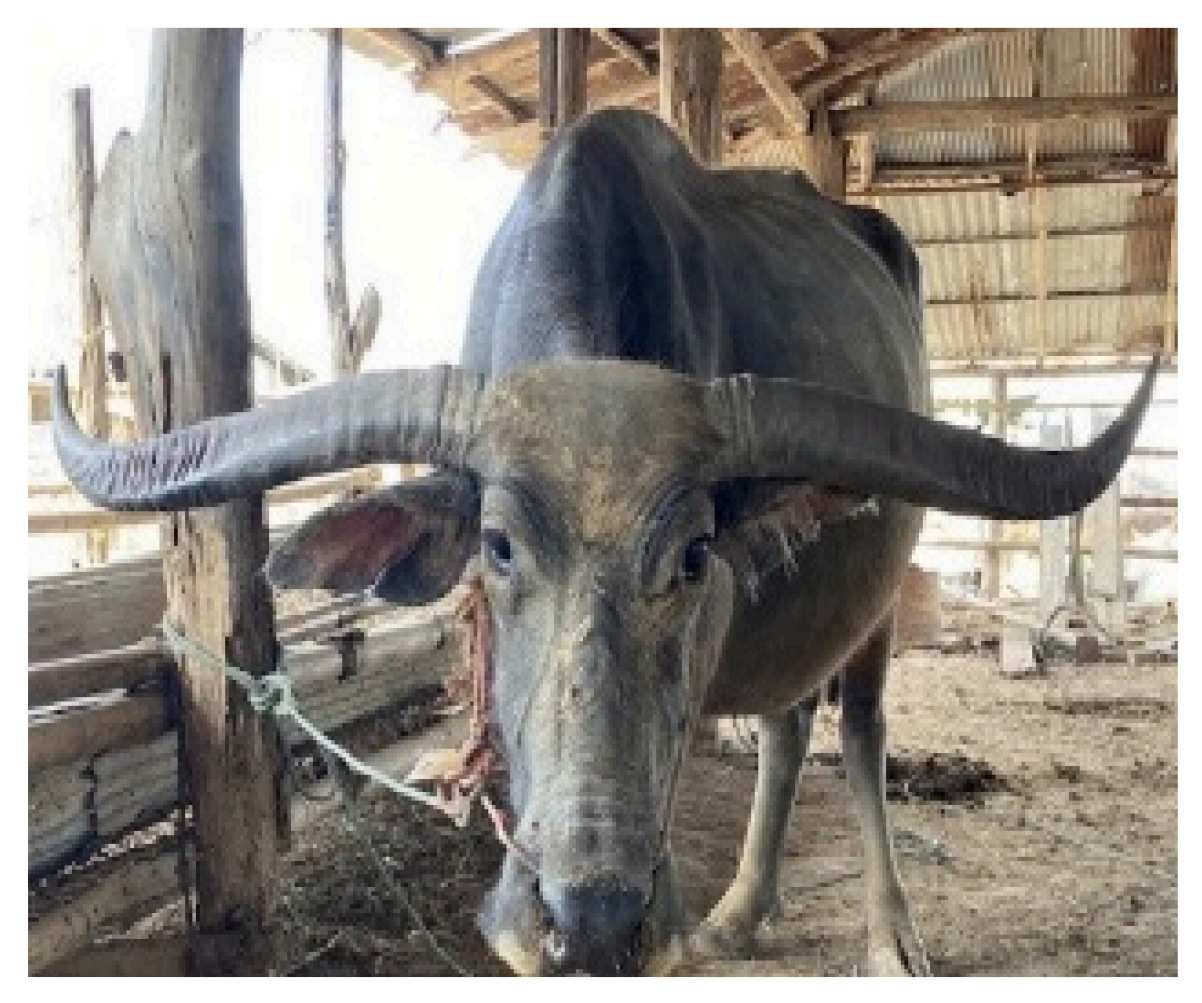	This horn type spreads widely, with a broad lateral flare and moderate outward curvature. Thick at the base and sometimes slightly twisted.	Blunt 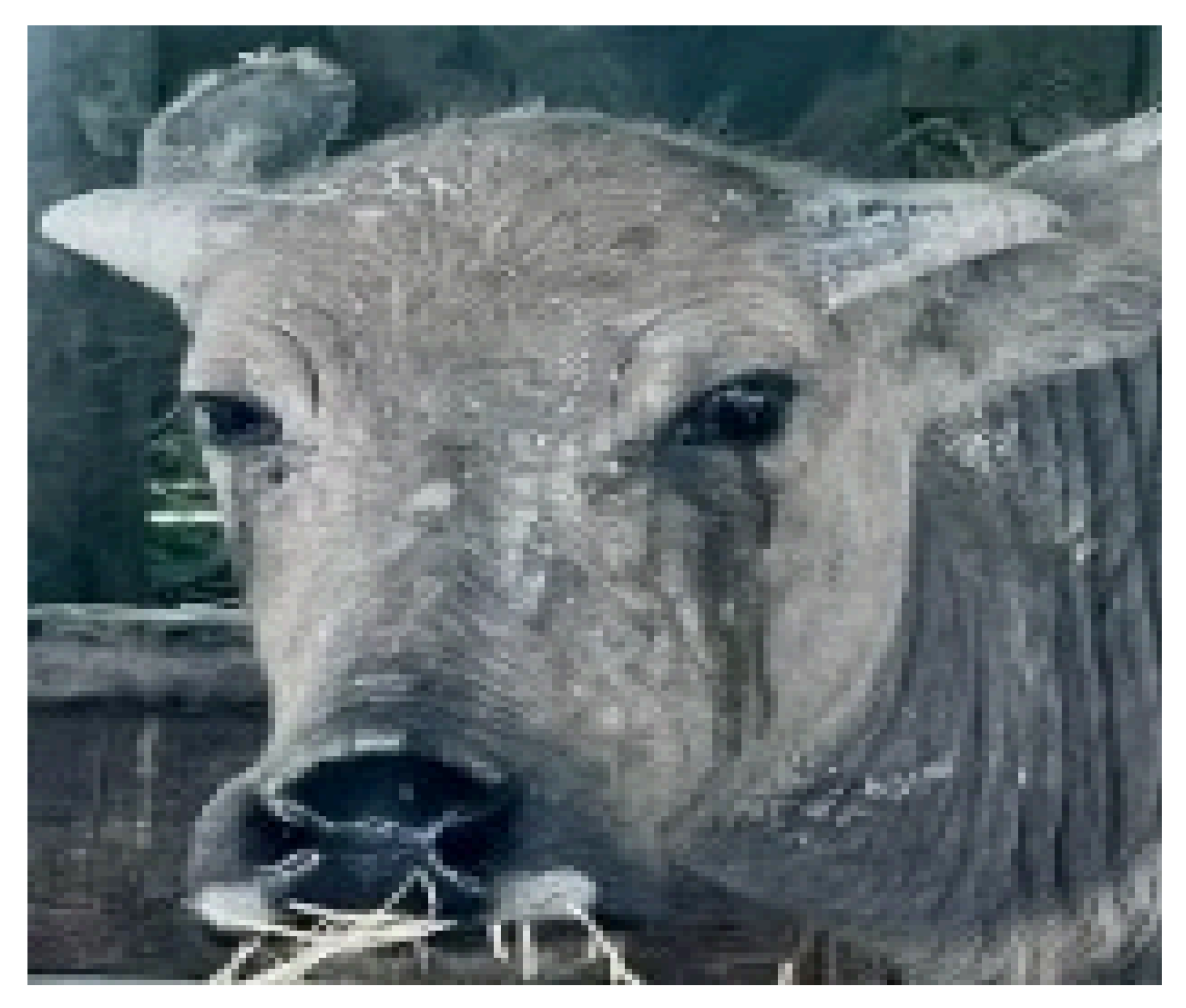	This horn type is short, thick, and nearly straight, with minimal tapering. Its blunt, dense structure reflects reduced elongation.
Stack 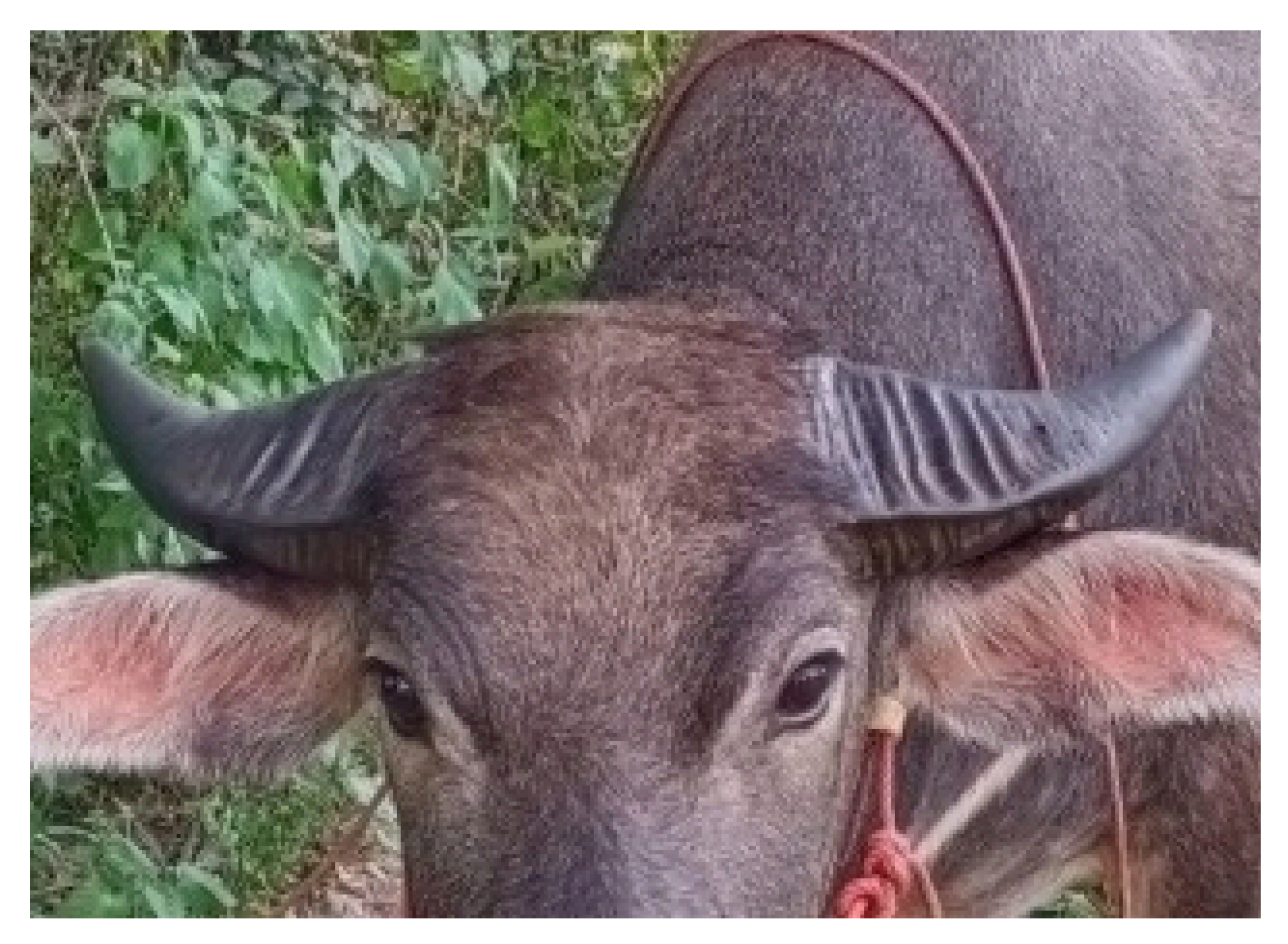	This horn type exhibits overlapping or layered curvatures, creating a stacked appearance with irregular bends or ridges.	Deform 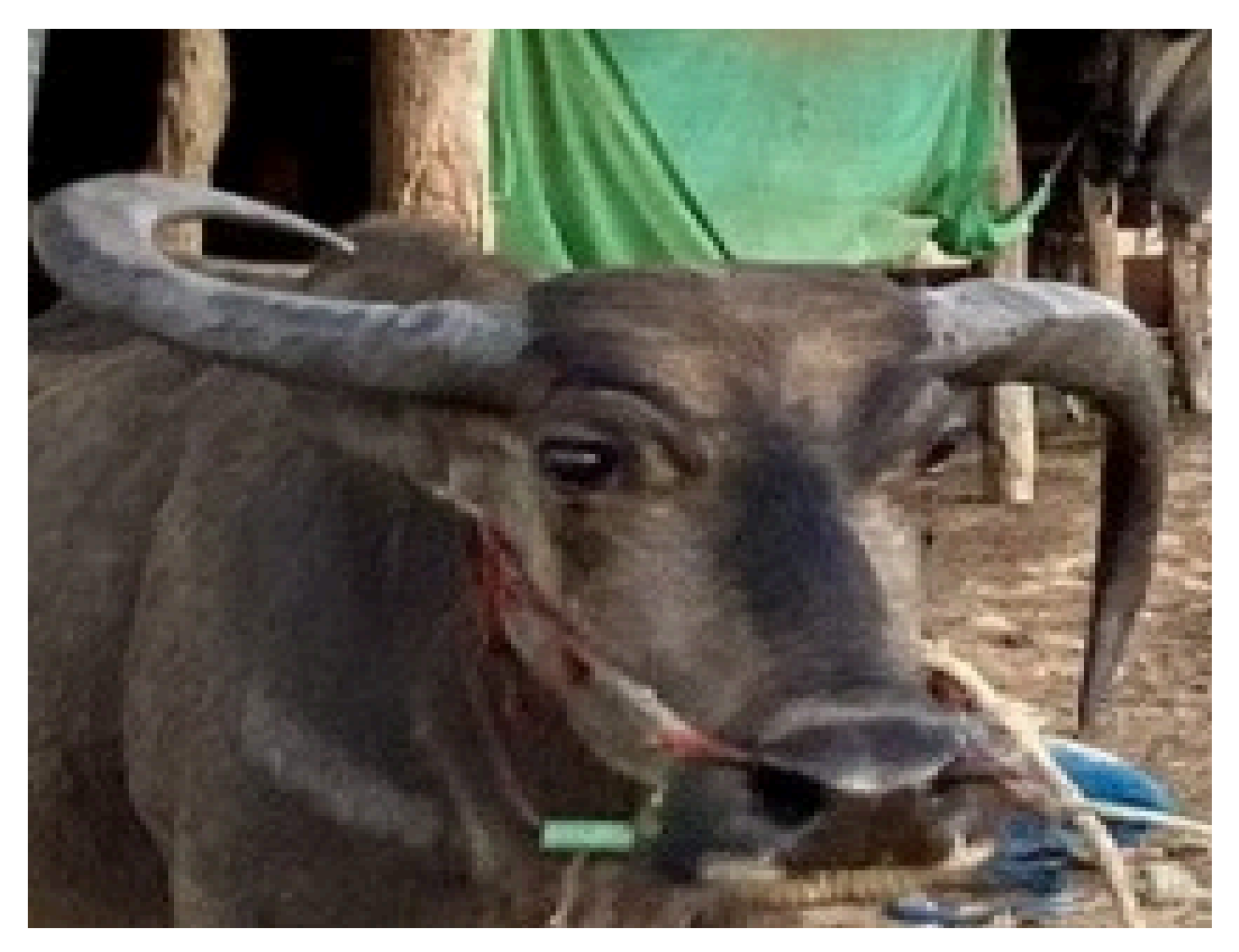	This horn type exhibits asymmetrical horn deformity, with one horn growing upward and the other downward. It is regarded as an abnormal horn formation rather than a natural morphotype.

**Table 3:** LS-means ^1^ (SE) of the body conformation characteristics of Thai swamp buffaloes in seven regions.

Body Measurement ^2^	Sex (S)	N	Region (R) ^3^							RxS Pval
			C	N1	N2	NE1	NE2	S1	S2	
1. Body depth	F	665	82.1 (3.9)	70.4 (2.2) ^a^	70.5 (2.8)	77.7 (2.0)	71.6 (2.6) ^a^	72.1 (5.4)	72.8 (3.4)	0.0083
	M	133	85.4 (4.8)	74.1 (2.6) ^b^	70.3 (3.4)	77.4 (2.5)	79.8 (3.0) ^b^	75.7 (7.7)	72.1 (3.5)	
2. Back height	F	665	131.8 (3.2) ^a^	120.5 (2.1) ^a^	121.3 (2.5) ^a^	133.2 (2) ^a^	125.9 (2.3) ^a^	121.0 (4.4)	116.3 (2.8)	<0.0001
	M	133	141.5 (3.9) ^b^	125.4 (2.4) ^b^	126.8 (2.9) ^b^	137.0 (2.4) ^b^	137.5 (2.6) ^b^	125.3 (6.4)	116.0 (2.9)	
3. Hip height	F	665	137.0 (3.6) ^a^	123.1 (2.4) ^a^	123.8 (2.9) ^a^	133.1 (2.4) ^a^	124.7 (2.6) ^a^	124.8 (5.0)	119.4 (3.1)	<0.0001
	M	133	146.6 (4.4) ^b^	127.7 (2.7) ^b^	129.9 (3.3) ^b^	135.8 (2.7) ^b^	137.2 (2.9) ^b^	128.6 (7.0)	120.1 (3.2)	
4. Tail head height	F	665	126.7 (3.5) ^a^	116.2 (2.2) ^a^	115.9 (2.6) ^a^	126.4 (2.1) ^a^	119.7 (2.4) ^a^	115.8 (4.8)	110.6 (3.0)	<0.0001
	M	133	137.7 (4.3) ^b^	120.6 (2.5) ^b^	121.1 (3.1) ^b^	132.1 (2.4) ^b^	132.3 (2.7) ^b^	116.7 (6.8)	112.8 (3.1)	
5. Shoulder height	F	665	136.1 (4.0) ^a^	120.2 (2.8) ^a^	121.5 (3.2) ^a^	137.2 (2.7)	125.2 (2.9) ^a^	124.1 (5.7)	119.3 (3.5)	<0.0001
	M	133	146.2 (4.8) ^b^	125.2 (3.1) ^b^	127.2 (3.7) ^b^	138.5 (3.0)	143.6 (3.2) ^b^	129.4 (7.7)	120.6 (3.6)	
6. Point of shoulder height	F	665	97.2 (6.3) ^a^	85.3 (3.9)	86.5 (4.8) ^a^	99.4 (3.7)	87.5 (4.3) ^a^	83.3 (8.9)	76.7 (5.3)	<0.0001
	M	133	112.8 (6.9) ^b^	87.0 (4.1)	91.6 (5.1) ^b^	102.5 (4.0)	93.3 (4.5) ^b^	84.9 (10.4)	78.7 (5.4)	
7. Flank height	F	665	50.5 (1.6) ^a^	51.1 (1.1)	52.6 (1.3) ^a^	53.0(1.1) ^a^	52.3 (1.2) ^a^	49.0 (2.1)	44.3 (1.5)	<0.0001
	M	133	61.9 (2.4) ^b^	52.1 (1.4)	58.4 (1.8) ^b^	55.7 (1.4) ^b^	55.9 (1.5) ^b^	51.6 (4.1)	45.0 (1.6)	
8. Rear hock joint height	F	665	50.4 (1.7)	47.4 (1.1) ^a^	47.6 (1.3)	51.1 (1.0)	48.6 (1.2) ^a^	46.3 (2.4)	40.0 (1.5)	<0.0001
	M	133	53.2 (2.3)	48.6 (1.3) ^b^	53.2 (1.7)	52.2 (1.3)	52.0 (1.4) ^b^	48.4 (3.9)	41.5 (1.6)	
9. Body length	F	666	145.5 (5.9) ^a^	135.1 (4.0) ^a^	137.3 (4.7)	145.8 (3.9)	129.3 (4.3) ^a^	137.7 (8.2)	132.1 (5.1) ^a^	<0.0001
	M	133	158.3 (7.3) ^b^	143.2 (4.5) ^b^	135.2 (5.5)	144.0 (4.5)	152.3 (4.8) ^b^	134.9 (11.7)	139.6 (5.3) ^b^	
10. Back length	F	666	144.6 (6.2) ^a^	112.8 (4.1)	120.0 (4.8)	130.8 (3.9)	116.7 (4.4) ^a^	128.6 (8.7) ^a^	119.3 (5.3)	<0.0001
	M	133	161.7 (7.2) ^b^	110.1 (4.5)	121.3 (5.5)	130.9 (4.4)	130.0 (4.8) ^b^	152.8 (11.4) ^b^	122.1 (5.4)	
11. Shoulder length	F	665	142.4 (20.8)	58.2 (11.2) ^a^	69.2 (14.8)	98.7 (9.9)	84.6 (13.2) ^a^	135.1 (29.5)	45.5 (17.1)	<0.0001
	M	132	149.0 (21.1)	65.3 (11.3) ^b^	70.6 (15.0)	99.8 (10.1)	101.8 (13.4) ^b^	139.1 (30.3)	50.0 (17.1)	
12. Shoulder width	F	664	58.0 (5.7)	35.8 (3.9) ^a^	44.3 (4.6)	52.7 (3.9)	43.1 (4.0) ^a^	40.0 (8.1)	37.3 (4.8)	0.0490
	M	131	59.7 (6.1)	41.0 (4.1) ^b^	45.7 (4.8)	53.6 (4.0)	48.9 (4.2) ^b^	45.1 (9.0)	39.9 (4.8)	
13. Hip width	F	664	61.0 (2.6) ^a^	46.8 (1.4) ^a^	44.0 (1.9)	53.4 (1.3)	51.2 (1.7) ^a^	52.7 (3.6)	43.0 (2.2)	0.0038
	M	132	64.8 (3.0) ^b^	48.9 (1.6) ^b^	45.1 (2.2)	55.3 (1.6)	58.1 (1.9) ^b^	53.8 (4.8)	44.7 (2.3)	
14. Heart girth	F	665	204.4 (8.1)	178.5 (5.4)	183.8 (6.4)	202.6 (5.2)	187.1 (5.8)	183.1 (11.4)	175.5 (7.0)	0.4119
	M	133	212.8 (9.8)	185.2 (6.0)	185.2 (7.4)	204.2 (5.9)	199.0 (6.4)	186.8 (15.7)	180.7 (7.3)	
15. Fr. cannon circum.	F	658	36.1 (2.5) ^a^	32.9 (1.4) ^a^	31.9 (1.8) ^a^	35.2 (1.3)	32.9 (1.6) ^a^	29.9 (3.5)	29.7 (2.1) ^a^	<0.0001
	M	131	39.4 (2.7) ^b^	35.3 (1.5) ^b^	34.7 (1.9) ^b^	34.8 (1.4)	37.5 (1.7) ^b^	33.6 (4.1)	32.9 (2.1) ^b^	
16. Fr. hoof circum.	F	661	43.1 (3.9)	48.4 (2.2) ^a^	48.6 (2.8) ^a^	48.9 (2) ^a^	48.8 (2.6) ^a^	45.3 (5.5)	47.0 (3.3) ^a^	<0.0001
	M	130	44.4 (4.4)	51.1 (2.4) ^b^	51.9 (3.1) ^b^	45.4 (2.2) ^b^	55.5 (2.7) ^b^	51.8 (6.7)	50.9 (3.4) ^b^	
17. Fr. hoof length	F	663	26.7 (1.7) ^a^	21.8 (1.0) ^a^	21.7 (1.3) ^a^	24.4 (1.0)	22.2 (1.2) ^a^	21.5 (2.4)	20.6 (1.5)	0.0051
	M	132	29.8 (2.0) ^b^	23.4 (1.1) ^b^	23.7 (1.4) ^b^	24.4 (1.1)	25.6 (1.3) ^b^	23.9 (3.1)	21.4 (1.5)	
18. Fr. fetlock circum.	F	665	15.9 (1.7)	8.7 (0.9)	9.2 (1.2)	12.0 (0.8)	11.8 (1.1)	16.7 (2.4)	8.1 (1.4)	0.0969
	M	130	17.0 (1.8)	9.5 (1.0)	8.7 (1.3)	12.9 (0.9)	13.2 (1.1)	17.3 (2.7)	9.1 (1.4)	
19. Fr. hoof width	F	665	16.1 (1.0)	12.5 (0.6)	13.1 (0.7)	14.4 (0.5)	12.1 (0.7)	14.4 (1.4)	11.1 (0.9)	0.3322
	M	130	18.0 (1.3)	14.6 (0.7)	13.7 (0.9)	15.2 (0.7)	13.9 (0.8)	15.2 (2.1)	12.7 (0.9)	
20. Tail switch length	F	662	46.7 (4.2)	39.0 (2.7)	40.0 (3.3)	43.4 (2.6)	32.9 (3.0)	37.3 (5.9)	33.6 (3.7)	0.2517
	M	131	48.8 (5.2)	36.2 (3.1)	42.1 (3.8)	39.5 (3.0)	29.8 (3.3)	29.1 (8.2)	33.2 (3.8)	
21. Forehead width	F	666	23.0 (0.8)	20.2 (0.5)	20.6 (0.6)	22.8 (0.5) ^a^	21.8 (0.6) ^a^	20.9 (1.1)	19.9 (0.7)	0.0154
	M	132	22.9 (1.2)	20.1 (0.6)	20.9 (0.8)	24.0 (0.7) ^b^	24.1 (0.7) ^b^	18.5 (2.0)	19.9 (0.8)	
22. Interocular distance	F	664	16.7 (1.2)	16.5 (0.7)	17 (0.9)	20.1 (0.7)	19.4 (0.8)	20 (1.7)	17.3 (1.1)	0.3402
	M	131	17.6 (1.5)	16.9 (0.8)	17.5 (1.1)	22 (0.8)	21.4 (1.0)	21.5 (2.5)	18.4 (1.1)	
23. Head length	F	666	52.2 (2.5)	41.8 (1.4)	44.2 (1.8)	49.4 (1.3)	46.6 (1.6)	51 (3.5)	44.6 (2.1)	0.0011
	M	132	54.8 (2.8)	44.1 (1.5)	46.9 (2.0)	51.8 (1.5)	53.2 (1.8)	58.8 (4.3)	47.4 (2.1)	
24. Upper horn length	F	666	58.2 (3.2)	45.7 (2.2) ^a^	46.3 (2.6)	54.7 (2.2) ^a^	48.2 (2.3) ^a^	50.0 (4.4)	46.6 (2.8) ^a^	<0.0001
	M	132	60.0 (4.1)	49.4 (2.6) ^b^	44.9 (3.1)	59.3 (2.6) ^b^	59.6 (2.7) ^b^	45.0 (6.8)	52.7 (3.0) ^b^	
25. Horn length from base	F	665	61.1 (2.5)	56.4 (1.7)	55.4 (2.0)	59.2 (1.7) ^a^	53.4 (1.9) ^a^	49.1 (3.5)	54.7 (2.3) ^a^	0.0002
	M	132	66.8 (3.8)	59.5 (2.2)	54.4 (2.8)	64.1 (2.2) b	64.3 (2.4) ^b^	41.3 (6.4)	61.4 (2.6) ^b^	
26. Horn tip distance	F	666	67.4 (3.9)	59.9 (2.9)	60.9 (3.2)	66.5 (2.8)	61.7 (3.1)	51.4 (5.2)	61.5 (3.7)	0.5902
	M	133	78.9 (6.2)	67.8 (3.8)	65.8 (4.6)	75.7 (3.7)	76.0 (3.9)	62.9 (10.9)	73.2 (4.1)	
27. Horn base circum.	F	659	26.2 (1.3)	24.2 (0.9)	23.7 (1.1)	26.9 (0.9)	25.0 (1.0)	23.3 (1.8)	22.5 (1.2)	0.1405
	M	130	33.0 (1.7)	30.0 (1.1)	29.1 (1.3)	33.9 (1.1)	32.9 (1.1)	34.3 (2.9)	29.5 (1.2)	
28. Horn base width	F	666	10.8 (0.5) ^a^	8.6 (0.4) ^a^	8.5 (0.4) ^a^	9.9 (0.4) ^a^	9.1 (0.4) ^a^	7.7 (0.7) ^a^	8.1 (0.5) ^a^	0.0048
	M	133	12.2 (0.7) ^b^	10.8 (0.4) ^b^	10.3 (0.5) ^b^	12.2 (0.4) ^b^	12.4 (0.5) ^b^	12.1 (1.2) ^b^	10.7 (0.5) ^b^	
29. Horn base thickness	F	666	6.6 (0.4) ^a^	5.6 (0.2) ^a^	5.2 (0.3) ^a^	6.3 (0.2) ^a^	5.8 (0.3) ^a^	5.5 (0.5) ^a^	4.3 (0.3) ^a^	0.0021
	M	132	8.2 (0.5) ^b^	7.0 (0.3) ^b^	6.0 (0.4) ^b^	7.5 (0.3) ^b^	8.0 (0.3) ^b^	7.4 (0.9) ^b^	5.3 (0.4) ^b^	
30. Tail length	F	666	94.1 (4.4)	76.5 (3.0)	81.3 (3.5)	89.9 (3.0)	78.7 (3.2) ^a^	80.4 (6.1)	72.3 (3.8)	0.0279
	M	132	100.4 (5.3)	79.6 (3.4)	84.4 (4.1)	91.5 (3.4)	88.6 (3.5) ^b^	89.2 (8.5)	73.5 (4.0)	

^1^ LS-means represent means adjusted for the fixed effects of age, while accounting for random variation due to province and farm; LS-means (SE) followed by different superscripts are significantly difference between sex within the same region at *p* < 0.05; ^2^ All traits were measured in centimeters; ^3^ C = Central, N1 = Upper North, N2 = Lower North, NE1 = Upper Northeast, NE2 = Lower Northeast, S1 = Upper South, S2 = Lower South; Fr. = Front; circum. = circumference.

**Table 4:** Genetic diversity indices and Wright’s F-statistics for Thai swamp buffalo population across geographic regions based on genome-wide SNP data ^1^.

Region	N	***p***	***q***	Ho	He	Chisq	Pval	PIC	H_I_	H_S_	H_T_	F_ST_	F_IS_	F_IT_
Central	43	0.757	0.243	0.322	0.330	1.501	0.446	0.266	0.322	0.330	0.334	0.0071	0.0249	0.0414
Upper North	74	0.760	0.240	0.313	0.327	1.519	0.448	0.264	0.313	0.327	0.334	0.0037	0.0453	0.0383
Lower North	76	0.757	0.243	0.329	0.332	1.265	0.467	0.268	0.329	0.332	0.334	0.0041	0.0085	0.0386
Upper Northeast	116	0.754	0.246	0.331	0.335	1.295	0.466	0.270	0.331	0.335	0.334	0.0029	0.0106	0.0375
Lower Northeast	113	0.759	0.241	0.328	0.329	1.228	0.470	0.266	0.328	0.329	0.334	0.0038	0.0051	0.0380
Upper South	14	0.773	0.227	0.335	0.306	0.890	0.525	0.246	0.335	0.306	0.334	0.0264	−0.0935	0.0596
Lower South	38	0.782	0.218	0.285	0.296	1.420	0.486	0.238	0.285	0.296	0.334	0.0536	0.0347	0.0797
Average	474	0.755	0.245	0.323	0.334	2.645	0.373	0.270	0.323	0.322	0.334	0.0170	0.0220	0.0386

^1^ N = number of buffalo genotyped; *p*, *q* = allele frequencies of the major and minor alleles across SNP loci; Ho = observed heterozygosity; He = expected heterozygosity under HWE; Chisq = Chi-square statistic for HWE test; Pval = probability associated with the Chi-square test; PIC = polymorphic information content; H_I_ = mean observed heterzygosity within individuals; H_S_ = expected heterozygosity within populations; H_T_ = expected heterozygosity in the total population; F_ST_ = genetic differentiation among populations; F_IS_ = deviation from random mating within populations; F_IT_ = overall inbreeding coefficient of individuals relative to the total population.

**Table 5:** Pairwise Wright’s F-statistics among seven regional populations of Thai swamp buffalo: (A) pairwise F_ST_ estimates and (B) pairwise F_IS_ (above diagonal) and F_IT_ (lower diagonal) estimates ^1^.

(A)								(B)							
	C	N1	N2	NE1	NE2	S1	S2		C	N1	N2	NE1	NE2	S1	S2
C	.	0.0084	0.0099	0.0076	0.0091	0.0326	0.0608	C	.	0.0465	0.0230	0.0208	0.0170	0.0154	0.0419
N1		.	0.0059	0.0046	0.0056	0.0289	0.0562	N1	0.0545	.	0.0333	0.0293	0.0263	0.0360	0.0510
N2		.	.	0.0046	0.0057	0.0292	0.0577	N2	0.0327	0.0390	.	0.0150	0.0118	0.0050	0.0255
NE1		.	.	.	0.0039	0.0285	0.0566	NE1	0.0283	0.0337	0.0196	.	0.0123	0.0080	0.0226
NE2		.	.	.	.	0.0303	0.0579	NE2	0.0260	0.0318	0.0174	0.0162	.	0.0028	0.0186
S1		.	.	.	.	.	0.0794	S1	0.0474	0.0638	0.0340	0.0363	0.0331	.	0.0190
S2		.	.	.	.	.	.	S2	0.1001	0.1043	0.0817	0.0779	0.0754	0.0968	.

^1^ C = Central, N1 = Upper North, N2 = Lower North, NE1 = Upper Northeast, NE2 = Lower Northeast, S1 = Upper South, S2 = Lower South.

**Table 6:** Number, length, and distribution of runs of homozygosity (ROH) across regions based on genome-wide SNP data ^1^.

Region	N	Number of ROH			Length of ROH (Mb)			FROH ^2^	Distribution of ROH Length (Mb) Cumulative Length of ***ROH*** (Mb)					
		Total	Avg (SD)	Unique	Total	Avg (SD)	Unique		1–2	2–4	4–8	8–12	12–16	>16
Central	43	1323	30.8 (16.80)	230	3938	91.6 (82.09)	1467	0.045	611	473	179	35	17	8
Upper North	74	2549	34.5 (15.68)	145	7204	97.4 (77.42)	1871	0.048	1211	937	294	71	19	17
Lower North	76	2199	28.9 (14.38)	197	5667	74.6 (70.14)	1766	0.037	1134	821	177	46	14	7
Upper Northeast	116	3080	26.6 (11.85)	159	7145	61.6 (51.48)	1829	0.030	1691	1165	173	35	10	6
Lower Northeast	113	2922	25.9 (9.71)	146	6946	61.5 (54.19)	1818	0.030	1653	1047	155	41	18	8
Upper South	14	365	26.1 (3.79)	223	730	52.1 (9.72)	538	0.026	216	139	10	0	0	0
Lower South	38	2265	59.6 (13.86)	154	6837	179.9 (73.30)	1838	0.089	969	889	303	76	18	10
Total	474	14,703	31.0 (15.75)	60	38,466	81.2 (71.55)	1950	0.040	7485	5471	1291	304	96	56

^1^ N = number of buffalo with SNP typing; ROH *=* Runs of homozygosity; Avg = Average; SD = Standard deviation; Unique = Unique ROH; Mb = Megabase pair; FROH= Fraction of the genome in runs of homozygosity. ^2^ Autosomal chromosome length = 2023.7 Mb.

## Data Availability

Unidentified metadata, LD-pruned autosomal with standard QC SNP data are provided in Supplementary Materials. The complete SAS and R scripts deposited at http://abagkku.in.th/monchai. Any use for teaching, or public communication requires permission and acknowledgment to corresponding author. Original SNP datasets and maps are restricted and may be provided upon request within a research collaboration.

## Supplementary Materials

The following supporting information can be downloaded at: https://www.mdpi.com/article/10.3390/ani16172713/s1, Table S1: SNP QC Summary; Table S2: Bootstrap CI for pariwise F-statistics; Table S3: Cross entropy for every runs and k; Figure S1: ROH distribution by Taxa; Figure S2: ROH hotspot SNP by taxa; Figure S3: Unfiltered Recurrent ROH shared across taxa.

## Abbreviations

The following abbreviations are used in this manuscript:

SNP	Single Nucleotide Polymorphism
PCA	Principal Component Analysis
CDA	Canonical Discriminant Analysis
PIC	Polymorphism Information Content
UPGMA	Unweighted Pair Group Mathematical Average
sNMF	Sparse Non-negative Matrix Factorization
ROH	Runs of homozygosity
FROH	Fraction of the genome in runs of homozygosity
